# MicroRNA-27b-3p down-regulates *FGF1* and aggravates pathological cardiac remodelling

**DOI:** 10.1093/cvr/cvab248

**Published:** 2021-08-06

**Authors:** Guoqi Li, Yihui Shao, Hong Chang Guo, Ying Zhi, Bokang Qiao, Ke Ma, Jie Du, Yong Qiang Lai, Yulin Li

**Affiliations:** Beijing Anzhen Hospital, Capital Medical University, The Key Laboratory of Remodelling-Related Cardiovascular Diseases, Ministry of Education, Beijing Institute of Heart, Lung and Blood Vessel Diseases, Beijing 100029, China; Beijing Anzhen Hospital, Capital Medical University, The Key Laboratory of Remodelling-Related Cardiovascular Diseases, Ministry of Education, Beijing Institute of Heart, Lung and Blood Vessel Diseases, Beijing 100029, China; Beijing Anzhen Hospital, Capital Medical University, The Key Laboratory of Remodelling-Related Cardiovascular Diseases, Ministry of Education, Beijing Institute of Heart, Lung and Blood Vessel Diseases, Beijing 100029, China; Beijing Anzhen Hospital, Capital Medical University, The Key Laboratory of Remodelling-Related Cardiovascular Diseases, Ministry of Education, Beijing Institute of Heart, Lung and Blood Vessel Diseases, Beijing 100029, China; Beijing Anzhen Hospital, Capital Medical University, The Key Laboratory of Remodelling-Related Cardiovascular Diseases, Ministry of Education, Beijing Institute of Heart, Lung and Blood Vessel Diseases, Beijing 100029, China; Beijing Anzhen Hospital, Capital Medical University, The Key Laboratory of Remodelling-Related Cardiovascular Diseases, Ministry of Education, Beijing Institute of Heart, Lung and Blood Vessel Diseases, Beijing 100029, China; Beijing Anzhen Hospital, Capital Medical University, The Key Laboratory of Remodelling-Related Cardiovascular Diseases, Ministry of Education, Beijing Institute of Heart, Lung and Blood Vessel Diseases, Beijing 100029, China; Beijing Anzhen Hospital, Capital Medical University, The Key Laboratory of Remodelling-Related Cardiovascular Diseases, Ministry of Education, Beijing Institute of Heart, Lung and Blood Vessel Diseases, Beijing 100029, China; Beijing Anzhen Hospital, Capital Medical University, The Key Laboratory of Remodelling-Related Cardiovascular Diseases, Ministry of Education, Beijing Institute of Heart, Lung and Blood Vessel Diseases, Beijing 100029, China

**Keywords:** Heart failure, Genetics, Inflammation, Cardiac remodelling

## Abstract

**Aims:**

The heart undergoes pathological remodelling under increased stress and neuronal imbalance. MicroRNAs (miRNAs) are involved in post-transcriptional regulation of genes in cardiac physiology and pathology. However, the mechanisms underlying miRNA-mediated regulation of pathological cardiac remodelling remain to be studied. This study aimed to explore the function of endogenous microRNA-27b-3p (miR-27b-3p) in pathological cardiac remodelling.

**Methods and results:**

miR-27b-3p expression was elevated in the heart of a transverse aortic constriction (TAC)-induced cardiac hypertrophy mouse model. miR-27b-knockout mice showed significantly attenuated cardiac hypertrophy, fibrosis, and inflammation induced by two independent pathological cardiac hypertrophy models, TAC and Angiotensin II (Ang II) perfusion. Transcriptome sequencing analysis revealed that miR-27b deletion significantly down-regulated TAC-induced cardiac hypertrophy, fibrosis, and inflammatory genes. We identified fibroblast growth factor 1 (*FGF1*) as a miR-27b-3p target gene in the heart which was up-regulated in miR-27b-null mice. We found that both recombinant FGF1 (rFGF1) and inhibition of miR-27b-3p enhanced mitochondrial oxidative phosphorylation (OXPHOS) and inhibited cardiomyocyte hypertrophy. Importantly, rFGF1 administration inhibited cardiac hypertrophy and fibrosis in TAC- or Ang II-induced models and enhanced OXPHOS by activating PGC1α/β.

**Conclusions:**

Our study demonstrated that miR-27b-3p induces pathological cardiac remodelling and suggests that inhibition of endogenous miR-27b-3p or administration of FGF1 might have the potential to suppress cardiac remodelling in a clinical setting.

## Graphical Abstract

**Graphical Abstract:**
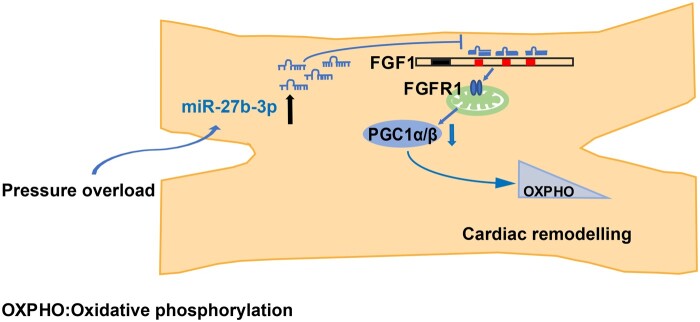

## 1. Introduction

Heart failure (HF) is a leading cause of death worldwide.^1^ HF is characterized by progressive myocardial hypertrophy, apoptosis, ventricular dilation, and fibrosis.^2,3^ During the early stages of cardiac hypertrophy, an increase in myocardial contractility is a short-term adaptive mechanism. Over time, however, persistent stress will result in ventricular dilation and decreased contractile function, eventually leading to HF.^4^ However, the molecular pathways that regulate this process are not fully understood.

Increasing evidence shows that microRNAs (miRNAs) are involved in post-transcriptional regulation of gene expression in cardiac physiology and pathology.^5^ Cardiac biological processes are tightly regulated by different non-coding RNAs to maintain cardiac homeostasis.^6^ miRNA levels change during pathological cardiac remodelling,^7^ and systemic or cardiac-specific knockout of selected miRNAs can affect this process.^8,9^ For example, miR-22-null mice show attenuated cardiac hypertrophy and remodelling in response to two independent stressors, isoproterenol infusion and calcineurin transgene activation.^10^ Additionally, loss of miR-155 prevents the progression of HF and substantially extends the survival of calcineurin transgenic mice.^11^ miR-27b is enriched in the heart^12^; moreover, serum levels of miR-27b-3p significantly increase in patients with left ventricular hypertrophy.^13,14^ In addition, cardiac-specific knockout of Smad4 aggravates cardiac hypertrophy and causes up-regulation of miR-27b.^15^ Since miR-27b-3p might be a key regulator of cardiomyocyte remodelling, it is important to elucidate its role in pathological cardiac remodelling.

In this study, we found that systemic knockout of miR-27b attenuates pathological cardiac remodelling, including cardiac hypertrophy, myocardial fibrosis, and inflammation, caused by transverse aortic constriction (TAC) and Angiotensin (Ang) II. We also found that *FGF1*, a target gene of miR-27b-3p, increases mitochondrial oxidative phosphorylation and that supplementation with recombinant FGF1 attenuates pathological cardiac remodelling caused by TAC and Ang II.

## 2. Methods

### 2.1 Generation of miR-27b knockout transgenic mice

The miR-27b knockout mice were generated by Transcription activator-like effector nuclease (TALEN)-mediated gene targeting at Cyagen Biosciences (Guangzhou, China). Briefly, the 3P mature sequence of mMiR-27b (mirBase Accession number: MIMAT0000126, mouse chromosome 8) was selected as the TALEN target site. TALEN mRNAs were generated by *in vitro* transcription and injected into fertilized eggs of C57BL/6 mice. mMiR-27b knockout (KO) founders were identified by PCR and Sanger sequencing analyses of genomic DNA isolated from mouse tail biopsies. Homozygous mMiR-27bKO mice were born from a heterozygous intercross. Finally, we obtained mMir27b KO mice carrying a 10-bp deletion (TAAGTTCTGC) using primers 5′-ATGACAGACAGATCCCTCCTATCTCC-3′ and 5′-TCAGCACG CTGTTTGCACTCTT-3′. The schematic diagram for the generation of the miR-27b KO mice is shown in Supplementary material online, *Figure S1A*. All mice were maintained in specific pathogen-free conditions in the Beijing Institute of Heart Lung and Blood Vessel Diseases. All animal studies were approved by the Animal Care and Utilisation Committee of Capital Medical University.

### 2.2 Animal models and echocardiography

All mouse experiments were performed according to the local relevant guidelines. All mice were kept under specific pathogen-free conditions in the Beijing Institute of Heart Lung and Blood Vessel Diseases and given free access to food and water. The investigation conformed to the Guide for the Care and Use of Laboratory Animals published by the US National Institutes of Health (NIH Publication No. 85-23, 1996, revised 2011; available from www.nap.edu/catalog/5140.html). miR-27b KO and wild-type (WT) C57Bl/6J mice (10–12 weeks old) were subjected to sham or TAC operation for eight weeks, as described before.^16^ Subsequently, mice were euthanized with pentobarbital administration (200 mg/kg, ip, once) (Sigma-Aldrich), and blood and hearts were collected for histological, protein, and RNA analyses. Pulsed-wave Doppler imaging was used for the non-invasive assessment of the pressure gradient across the aortic constriction generated by TAC or sham operation in mice.

Experiments were performed using miR-27b transgenic mice and WT littermates as controls. The mice were anaesthetized with 2% isoflurane and maintained with 1.5% isoflurane (RWD Life Science, Catalogue: R510-22, Shenzhen, China), and each mouse was infused with AngII (Sigma-Aldrich, Catalogue: 05-23-0101, Darmstadt, Germany) (1500 ng/kg/min) or saline for 14 days subcutaneously by implantation of an osmotic mini-pump (ALZET Model 1002D, DURECT, Cupertino, CA, USA) through a small pocket made in the skin between the scapulae. The echocardiography was carried out with the Vevo2100 High-Resolution Imaging System (Vevo2100; Visual Sonics, Toronto, ON, Canada) for each mouse after anaesthesia with 2% isoflurane. All measurements were performed for five consecutive cardiac cycles. The values of intraventricular septal (IVS) thickness, left ventricular internal dimension (LVID), and left ventricular posterior wall (LVPW) thickness at diastole and systole, as well as LV fractional shortening (FS) and LV ejection fraction (EF%) were calculated.

### 2.3 MiRNA mimics, inhibitor, or FGFR1-siRNA design and transfection

The miR-27b mimics was a short double-stranded RNA; the inhibitor miR-27b was a single-stranded antisense RNA against miRNAs with 2’-O-Me modification, in which a single-stranded RNA identical to mature miRNAs would be incorporated into the RNA-induced silencing complex to mimic the effects of endogenous miRNAs. Primary cardiomyocytes or H9c2 cells were transfected with a mixture containing 50 nM mimic or 50 nM inhibitor, 2 μL Lipofectamine 3000 regent, and 50 µL Opti-MEM. Forty-eight hours after transduction, cardiomyocytes or H9c2 cells were subjected to isoproterenol (ISO) or phenylephrine (PE). Cells transfected with negative control (NC) mimic or NC inhibitor were used as controls. The sequences of the miR mimic/inhibitor are provided in Supplementary material online, *Table S1*. HEK293T cells were transfected with 50 ng of the appropriate luciferase reporter construct and 50 nM miR-27b-3p mimic or NC mimic. HEK293T cells were harvested and lysed for luciferase assay 48 h after transfection. FGFR1-targeted small interfering RNA (siRNA) or negative control siRNA was purchased from Santa Cruz Biotechnology. H9c2 cells were seeded at a density of 2.0 × 10^5^/well in a six-well dish for 24 h until they reached approximately 80% confluence and washed. Serum-free medium was added. Transient transfection of siRNA (100 nM) was performed using Lipofectamine 3000 reagent as per the manufacturer’s instructions.

### 2.4 Luciferase reporter assay

Luciferase assays were performed on a Synergy4 microplate reader following manufacturer’s instructions (BioTek, Winooski, VT, USA). The NM_010197-3utr-(miR-27b-3p) with SV40-firefly-luciferase-MCS vector was purchased from GENECHEM and contained the full-length 3ʹ-UTR of *Mmu FGF1*. The NM_010197-3utr-(miR27b-3p)-mut with SV40-firefly-luciferase-MCS vector contained the 3ʹ- UTR of *Mmu FGF1* mutated by two-site-directed mutagenesis (943, 1415) (GENE, Catalogue: 00010294, Shanghai, China) or three-site-directed mutagenesis (943, 1415, 2938) (GENE, Catalogue: 00016834, Shanghai, China). A luciferase assay was performed as described previously^15,17^ to detect the binding ability of miR-27b-3p mimic or NC mimic with *FGF1* in HEK293T cells.

### 2.5 Western blot analysis

Protein extracts (40∼80 μg) were electrophoresed at 80 volts on 10% or 12% SDS/PAGE, and then transferred onto nitrocellulose filter membrane (PALL BioTrace, Port Washington, NY, USA). Immunoblotting was performed according to the manufacturer’s instructions using the following antibodies: brain natriuretic peptide (BNP, abcam), ANP32E (abcam), FGF1 (abcam), Smad3 (Cell Signalling Technology), Phospho-Smad3 (CST), GAPDH (Zhong Shan Golden Bridge ZSGB), PGC1α (abcam), PGC1β (abcam), FGFR1 (abcam), Mac-2(Santa Cruz Biotechnology), IL-1β (abcam, Catalogue: ab9722), and NF-κB p65 (abcam). A Bio-Rad Imager facility was used to detect the expression of proteins.

### 2.6 Regents or kits

The catalogue numbers and manufacturers of all reagents and antibodies have been provided in Supplementary material online, *Table S1*.

### 2.7 FGF1 intraperitoneal injection in mice that underwent pressure overload and Ang II infusion

KO and WT mice were injected intraperitoneally with rFGF1 (0.5 mg/kg) every other day for 8 weeks after TAC or 2 weeks after Ang II infusion. rFGF1, which is approved for clinical use,^18^ was provided by Dr. Zhi Feng Huang (School of Pharmaceutical Sciences and Centre for Structural Biology, Wenzhou Medical University, Zhejiang, China). There was no peptide/protein modification in rFGF1.

### 2.8 Histology and immunohistochemistry

Samples were fixed in 10% formalin, processed, and paraffin-embedded. Multiple sections (5 μm) were prepared and stained with haematoxylin and eosin for general morphological observation. For Sirius Red (Solarbio, Catalogue: G1470, Beijing, China) staining, sections were stained for 1 h. For wheat germ agglutinin (WGA) (CytoFlamma, Catalogue: RCS113, Korea) staining, sections were incubated with WGA at 37°C for 1 h after serum blocking. For immunocytochemistry staining, sections were incubated with primary antibody at 4°C overnight. The signals were detected using a biotinylated corresponding secondary antibody in combination with DAB Substrate (Thermo Fisher Scientific, Catalogue: 34002, Boston, MA, USA). Samples were visualized with a Nikon Eclipse 80i upright microscope (Nikon, Tokyo, Japan). For histological assessment of fibrosis, paraffin-embedded sections of the LV were stained with picrosirius red and the collagen content calculated as the percentage of fibrotic areas in each LV section. For IL-1β levels, IHC-positive areas were measured in 8–10 visual fields in each section from similar positions, averaged, and presented as a percentage of the LV section.

### 2.9 Establishment of mouse cardiomyocyte hypertrophy model

All procedures were performed in accordance with the Guide for the Care and Use of Laboratory Animals. Neonatal mice of 1–2 days old were euthanized by decapitation. Cardiomyocytes and cardiac fibroblasts were isolated from neonatal mice as previously reported.^19,20^ Briefly, the ventricles were excised, washed three times, and cut into small pieces in sterile PBS plus penicillin/streptomycin (PS), and then digested with Neonatal Heart Dissociation Kit in a CO_2_ incubator to keep the reaction temperature at 37°C. After digestion for 15 min, 10 mL PBS solution was added to terminate the reaction. The mixture was filtered and centrifuged at 1000 rpm for 10 min. After culturing the cells in DMEM in a cell incubator for 2 h, the supernatant was removed, and then cardiac myocyte medium with 10% FBS and 100 µM BrdU was added for primary cardiomyocytes; for primary cardiac fibroblasts, adherent cells were further cultured in DMEM with 10% FBS. After 48 h, the medium was replaced with a serum-free maintenance medium for follow-up experiments. Cardiomyocytes and cardiac fibroblasts were treated with PBS or 10 µM ISO or 200 µM PE for 48 h.

### 2.10 Measurement of oxygen consumption

Primary cardiomyocytes were layered on a XF24 plate. Oxygen consumption rates (OCRs) in differentiated cells treated (PBS and rFGF1(100 ng/mL) for 24 h were measured using an XF24 respirometer (Seahorse Bioscience, Santa Clara, CA, USA) and seahorse XF Cell Mito Stress Test Kit (Agilent Technologies, Catalogue: 103015-100, CA, USA). Basal oxygen consumption was determined along with oxygen consumption in the presence of drugs disrupting the mitochondrial respiratory chain: oligomycin (OL, ATP synthase inhibitor, 1 mM) and carbonyl cyanide-4-(trifluoromethoxy) phenylhydrazone (FCCP, uncoupler, 1 mM). Finally, mitochondrial respiration was blocked with rotenone (Rot, 1 mM).

### 2.11 Human heart samples

Human hypertrophic heart tissue samples were obtained from patients previously diagnosed with hypertrophic cardiomyopathy (HCM) undergoing septal myectomy. Control samples were obtained from normal-heart donor left ventricles. All specimens were processed immediately after surgery and stored at temperatures below −80°C, until total RNA and protein were extracted. All patients or their relatives gave written informed consent before operation. The local Institutional Review Board of the Institute of Biophysics and Anzhen Hospital approved all studies involving human heart tissue samples. The investigation conformed to the principles outlined in the Declaration of Helsinki. Detailed information, such as age, sex, HF severity, control and HCM groups, application of medications, comorbidities, and familial genetic diseases, is presented in Supplementary material online, *Table S2*.

### 2.12 RNA transcriptome sequencing and analysis

Eight weeks after TAC, total mRNA from miR-27b-null and WT mice was extracted with Trizol (Invitrogen, Carlsbad, CA, USA). Two micrograms of total RNA was used for first-strand cDNA synthesis with avian myeloblastosis virus reverse transcriptase (Promega, Catalogue: M5108, Madison, USA), according to manufacturer’s protocol. Special Taqman probes were used for cDNA synthesis of miRNAs and oligo-dT for mRNA. The products of RT reaction were used for qRT-PCR. The expression levels of target genes were determined with SYBR master mix (Thermo Fisher Scientific, Catalogue: 4309155, USA) and analysed by CFX-96 (Bio-Rad, Hercules, CA, USA) system. Transcript levels of mRNA or miRNA were compared using the relative Ct method, where the amount of target gene was normalized to the amount of endogenous control (GAPDH or U6) and presented as 2^−^^ΔCt^. For example, the miR-27b-3p expression was calculated according to the following formula: quantity = 2^-difference in (miR-27b-3p—U6)^. The list of primer sequences for mRNA is shown in Supplementary material online, *Table S1*.

### 2.13 ELISA

FGF1 concentration was measured using ELISA with specific anti-mouse ELISA kits (Bio-Techne, Catalogue: DY4686-05, MN, USA) from R&D Systems. Assays were performed per the manufacturer’s protocol and read at 450 nm in a microplate reader (BioTek, Winooski, VT, USA). The concentrations were calculated from six samples as mean ± standard deviation.

### 2.14 Statistics

**Figure 1:**
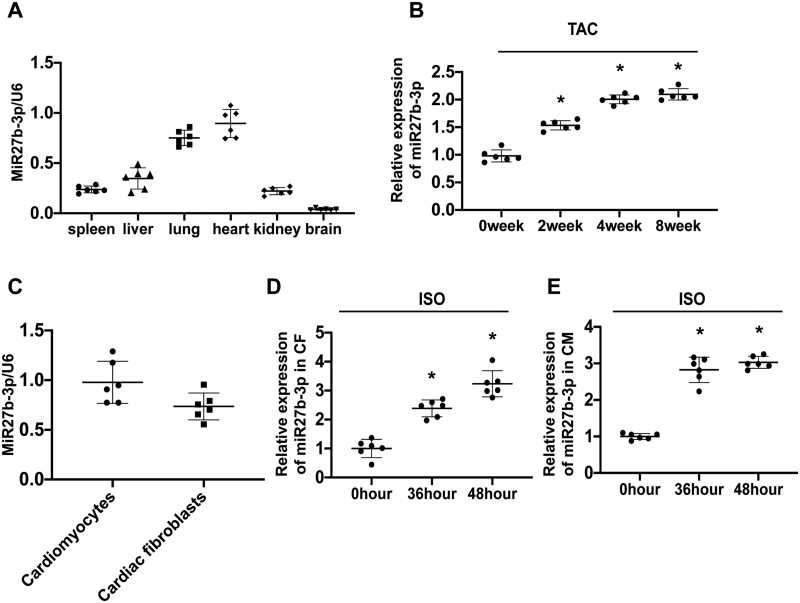
miR-27b-3p expression is elevated in cardiac hypertrophy. (*A*) Quantitative polymerase chain reaction (qPCR) of miR-27b-3p shows expression of miR-27b-3p in multiple organs of mice (*n* = 6). (*B*) miR-27b-3p is significantly up-regulated in whole heart tissue from mice 2, 4, and 8 weeks after TAC (*n* = 6). (*C*) miR-27b-3p is expressed in cardiomyocytes and cardiac fibroblasts from mice (*n* = 6). (*D*) miR-27b-3p is significantly up-regulated in ISO-treated isolated cardiac fibroblasts from mice (*n* = 6). (*E*) miR-27b-3p is significantly up-regulated in ISO-treated isolated cardiac myocytes from mice (*n* = 6). All data are shown as mean ± SD.*P < 0.05 compared to 0 week (*B*) or 0 h (*D, E*); for more than two groups, data were compared by one-way ANOVA with Bonferroni *post hoc* test (*B, D, E*).

**Figure 2:**
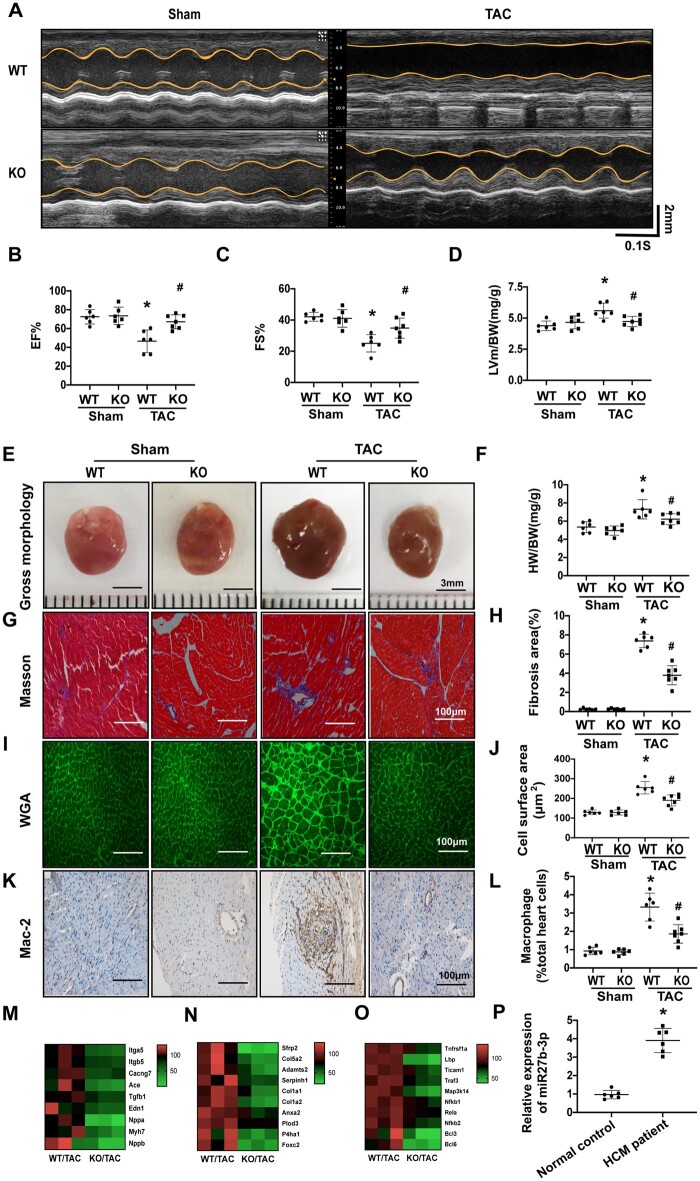
Loss of miR-27b attenuates cardiac pathological remodelling induced by pressure overload. (*A*) Representative examples of M-mode echocardiography of wild-type and miR-27b-null hearts under pressure overload or sham operation. Scale bar, 2 mm. (*B*–*C*) Quantification of left ventricular ejection fraction (EF%; *B*) and fraction shortening (FS%; *C*) in wild-type and miR-27b-null mice after TAC or sham operation. (*D*–*F*) Quantification of left ventricular mass-to-body weight (LV mass/BW; *D*) or heart weight-to-body weight ratio (HW/BW; *F*) 8 weeks after sham or TAC operation (WT *n* = 6, KO *n* = 6, and 7 for sham or TAC, respectively). The heart size (representative images of gross heart morphology; *E*) of wild-type and miR-27b-null mice after TAC or sham operation (WT *n* = 6; KO *n* = 7). Scale bar, 3 mm. (*G*–*H*) Representative images of heart sections from wild-type and miR-27b-null mice were stained with Masson’s trichrome after TAC or sham operation (WT *n* = 6, KO, *n* = 7; *G*). Quantification of the fibrosis area in wild-type and miR-27b-null mice after TAC or sham operation (WT *n* = 6, KO *n* = 7; *H*). (*I* and *J*) Representative images of heart sections from wild-type and miR-27b-null mice were immunostained with WGA after TAC or sham operation (WT *n* = 6, KO *n* = 7; *I*). Quantification of cell surface area in wild-type and miR-27b-null mice after TAC or sham operation (WT *n* = 6, KO *n* = 7; *J*). (*K* and *L*) Representative images of heart sections from wild-type and miR-27b-null mice were immunostained with Mac-2 antibody after TAC or sham operation (WT *n* = 6, KO *n* = 7; *K*). Quantification of MAC-2 expression in wild-type and miR-27b-null mice after TAC or sham operation (WT *n* = 6, KO *n* = 7; *L*). (*M*–*O*) Transcriptome analysis shows the expression of hypertrophic cardiomyopathy (HCM)-, TGF-beta signalling-, and NF-κB signalling-related genes. (*P*) miR-27b-3p is significantly up-regulated in left ventricular tissue from patients with cardiac hypertrophy (*n* = 6). Scale bar, 100 nm. All data are shown as mean ± SD.*P < 0.05 compared to WT/Sham; ^#^*P* < 0.05 compared to WT/TAC. For more than two groups, data were compared by two-way ANOVA with Bonferroni *post hoc* test. For two groups, data were compared by two-sample *t*-test.

**Figure 3:**
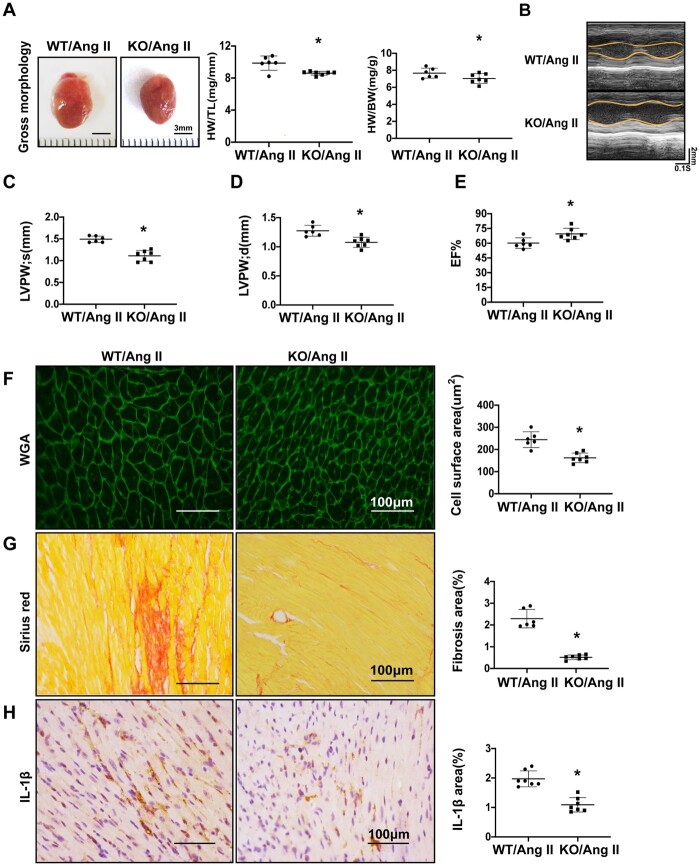
Loss of miR-27b attenuates cardiac pathological remodelling induced by Ang II perfusion. (*A*) Representative images of gross heart morphology of wild-type and miR-27b-null mice after Ang II infusion and quantification of heart weight/body weight ratio and heart weight/tibia length. (*B*) Representative examples of M-mode echocardiography of wild-type and miR-27b-null mice after Ang II infusion (WT *n* = 6, KO *n* = 7). (*C*–*E*) Quantification of left ventricular end-systolic posterior wall thickness (LVPWs; *C*), end-diastolic posterior wall thickness (LVPWd; *D*), and ejection fraction (EF%; *E*) (WT *n* = 6, KO *n* = 7). (*F*) Representative images of heart sections from wild-type and miR-27b-null mice 2 weeks after Ang II infusion. The heart sections were immunostained with WGA, in green. Scale bar, 100 μm. Quantification of the cross-sectional area of the cardiomyocytes is shown in a bar graph (WT *n* = 6, KO *n* = 7). (*G*) The heart sections were immunostained with Sirius Red. Scale bar, 100 μm. Quantification of the fibrosis area of the cardiomyocytes is shown in a bar graph (WT *n* = 6, KO *n* = 7). (*H*) Immunohistochemistry of heart sections with IL-1β antibody, in brown. Scale bar, 100 μm. Quantification of the positive area of the cardiomyocytes is shown in a bar graph (WT *n* = 6, KO *n* = 7). All data are shown as mean ± SD.*P < 0.05; data were compared by two-sample *t*-test.

**Figure 4:**
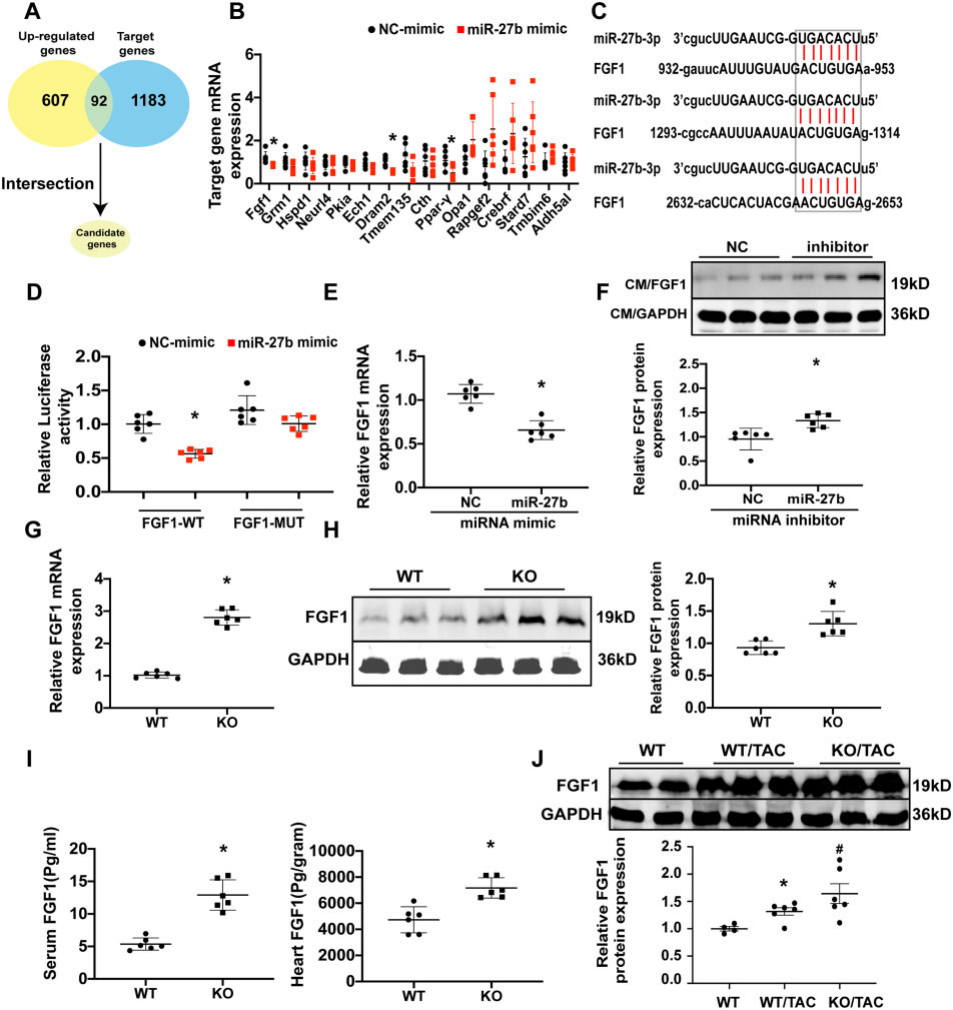
FGF1 serves as a target gene of miR-27b-3p. (*A*) Workflow to identify the candidate target genes of miR-27b-3p through our RNA-sequencing data and microRNA target prediction systems (www.microrna.org). (*B*) qPCR of top 16 candidate target genes in miR-27b-3p-mimic- or NC-mimic-treated H9c2 cells *in vitro*. (*C*) Predicted binding sites of FGF1 as a target of miR-27b-3p. (*D*) Luciferase assay confirmed miR-27b-3p association with FGF1 mRNA 3'-UTR. Constructs carrying the FGF1 3ʹ UTR or not (vector) were co-transfected with scramble NC mimic or miR-27b-3p mimic in 293T cells (*n* = 6). (*E*) qPCR analysis of the mRNA expression of FGF1 in NC-mimic- or miR-27b-3p-mimic-treated isolated cardiomyocytes (*n* = 6). (*F*) Western blot analysis of FGF1 protein expression in NC-inhibitor- or miR-27b-3p inhibitor-treated cardiomyocytes; GAPDH was used as a loading control (*n* = 6). (*G*) qPCR analysis of the mRNA expression of FGF1 in cardiac tissue of wild-type and miR-27b-null mice (*n* = 6). (*H*) Western blot analysis of FGF1 protein expression in whole heart of wild-type or miR-27b-null mice; GAPDH was used as a loading control (*n* = 6). (*I*) Concentration of FGF1 in heart or serum of wild-type and miR-27b-null mice assessed by ELISA (*n* = 6). (*J*) Western blot analysis of FGF1 protein expression in whole heart of wild-type and mir27b-3p-null mice after TAC; GAPDH was used as a loading control (*n* = 8). All data are shown as mean ± SD.*P < 0.05 compared to NC mimic (*B, D, E*) or NC inhibitor (*F*) or WT (*G, H, I, J*); ^#^*P* < 0.05 compared to WT/TAC. For two groups, data were compared by two-sample *t*-test. For more than two groups, data were compared by one-way ANOVA with Bonferroni *post hoc* test.

**Figure 5:**
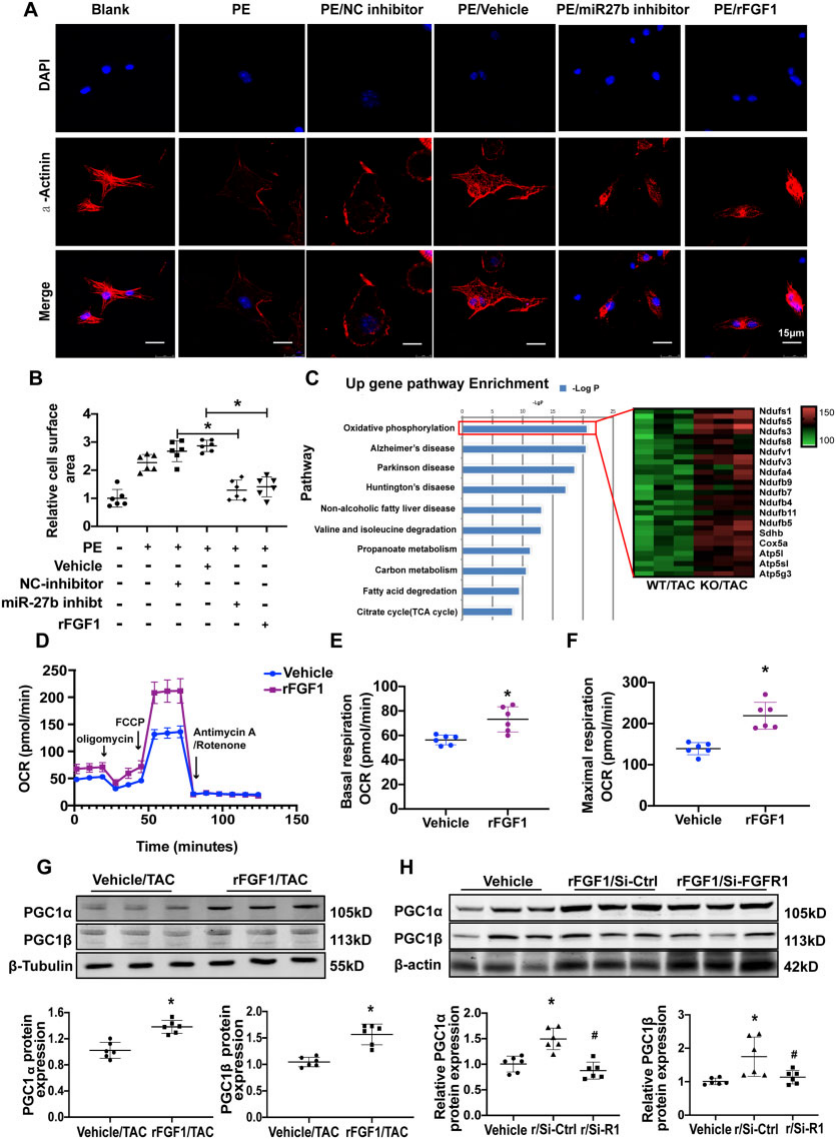
FGF1 up-regulates mitochondrial oxidative function and inhibits cardiomyocyte hypertrophy. (*A*) Isolated mouse neonatal cardiomyocytes (NCMs) were infected with rFGF1 or miR-27b-3p inhibitor, vehicle, and inhibitor-NC as control, respectively, and treated with PE. Cardiomyocytes were stained for α-actinin (red), and DAPI staining was used to visualize the nuclei (blue). Scale bar, 15 μm. (*B*) Quantification of fold change in mean cell surface area of α-actinin-immunostained cardiomyocytes (*n* = 6). (*C*) Pathway enrichment analysis using DAVID. Gene ontology (GO) annotation of the differentially expressed genes from the hearts of 8-week-old miR-27b-null and wild-type mice after TAC (*n* = 3). (*D*–*F*) In assays of mitochondrial respiration using extracellular flux analysis, basal oxygen consumption (*E*) and maximum oxygen consumption rate (*F*) (OCR) were measured in cultured NCMs, following treatment with vehicle or rFGF1 (both 100 ng/mL) for 24 h (*n* = 6). (*G*) Immunoblot for PGC-1α/PGC-1β in the heart from vehicle or rFGF1-treated mice 8 weeks after TAC (*n* = 6). (*H*) Immunoblot for PGC-1α/PGC-1β in H9c2 cells treated with vehicle or rFGF1 and rFGF1/siFGFR1 for 24 h (*n* = 6). All data are shown as mean ± SD.*P < 0.05 compared to Vehicle (*E, F*) or Vehicle/TAC (*G*); ^#^*P* < 0.05 compared to rFGF1/Si-Ctrl (*H*). R/Si-control means rFGF1+control-SiRNA, and R/Si-R1 means rFGF1+FGFR1-SiRNA. For two groups, data were compared by two-sample *t*-test; for more than two groups, data were compared by one-way ANOVA with Bonferroni *post hoc* test.

**Figure 6:**
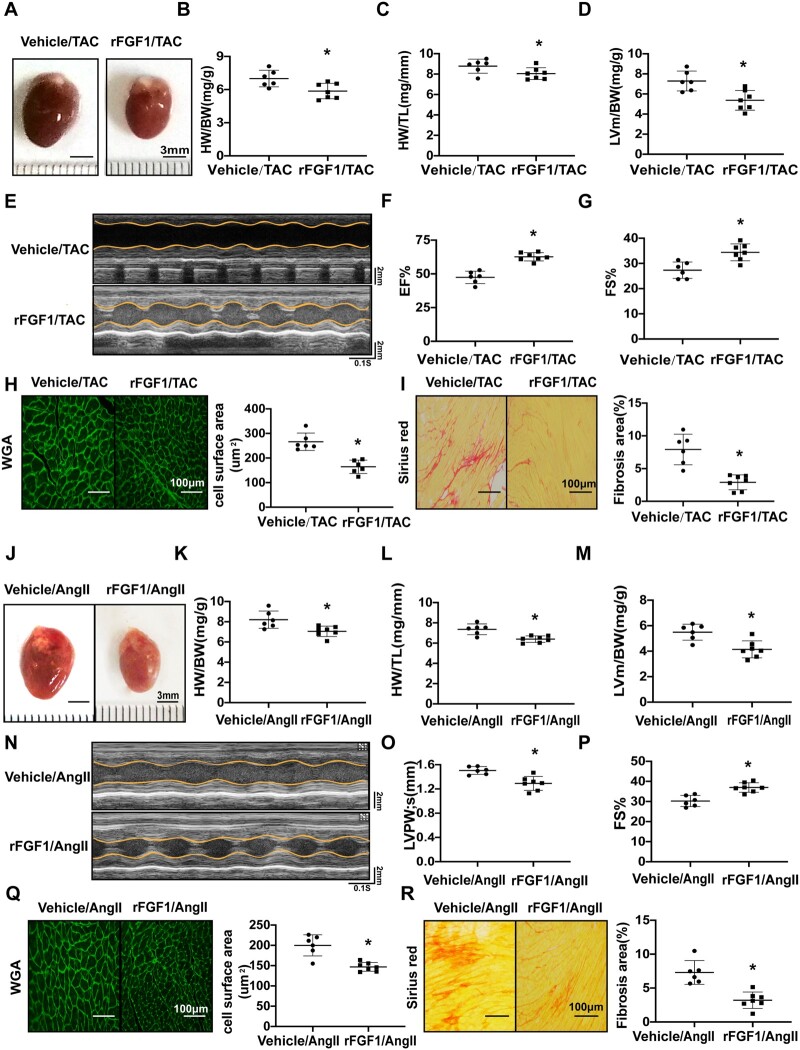
FGF1 attenuates the pathological cardiac remodelling induced by TAC and Ang II. (*A*) Representative images of gross heart morphology of vehicle- and FGF1-treated mice after TAC. Scale bar, 3 mm (Vehicle *n* = 6, FGF1-treated *n* = 7). (*B*–*C*) Quantification of heart weight/body weight ratio (HW/BW; *B*) and heart weight/tibia length (HW/TL; *C*) (Vehicle *n* = 6, FGF1-treated *n* = 7). *D*, Quantification of LV mass/BW (*D*) after TAC (Vehicle *n* = 6, FGF1-treated *n* = 7). (*E*–*G*) Representative examples of M-mode echocardiography of vehicle- and FGF1-treated hearts subjected to pressure overload (*E*). Quantification of left ventricular EF% (*F*) and FS% (*G*) (Vehicle *n* = 6, FGF1-treated *n* = 7). (*H*) Representative images of heart sections from vehicle- and FGF1-treated mice after TAC. Sections were immunostained with WGA, in green. Scale bar, 100 μm. Quantification of the cross-sectional area of cardiomyocytes is shown in a bar graph (Vehicle *n* = 6, FGF1-treated *n* = 7). (*I*) The heart sections were immunostained with Sirius Red (scale bar, 100 μm). Quantification of the fibrosis area of cardiomyocytes is shown in a bar graph (Vehicle *n* = 6, FGF1-treated *n* = 7). (*J*) Representative images of gross heart morphology of vehicle- and FGF1-treated mice after Ang II infusion (Vehicle *n* = 6, FGF1-treated *n* = 7). Scale bar, 3 mm. (*K* and *L*) Quantification of HW/BW (*K*) and HW/TL (*L*) (Vehicle *n* = 6, FGF1-treated *n* = 7). (*M*) Quantification of left ventricular LV mass/BW after Ang II infusion (Vehicle *n* = 6, FGF1-treated *n* = 7). (*N*–*P*) Representative examples of M-mode echocardiography of vehicle- and FGF1-treated hearts subjected to Ang II infusion. Quantification of LVPWs (*O*) and FS% (*P*) (Vehicle *n* = 6, FGF1-treated *n* = 7). (*Q* and *R*) Representative images of heart sections from vehicle- and FGF1-treated mice after TAC. Sections were immunostained with WGA, in green. Quantification of the cross-sectional area of cardiomyocytes is shown in a bar graph (*Q*). The heart sections were immunostained with Sirius Red, and quantification of the fibrosis area of the cardiomyocytes is shown in a bar graph (*R*) (Vehicle *n* = 6, FGF1-treated *n* = 7). Scale bar, 100 μm. All data are shown as mean ± SD.*P < 0.05 compared to Vehicle/TAC (*B, C, D, F, G, H, I*) or Vehicle/Ang II (*K, L, M, O, P, Q, R*). Data were compared by two-sample *t*-test.

Continuous variables are presented as mean±SEM, and categorical variables are presented as number and percentage. Differences between two groups were evaluated using the two-sample *t*-test or χ2 test. Specifically, the data in *Figures 2P*, *3A, C–H*, *4B, D–I, 5E–G, 6B–I, K–R*, and Supplementary material online, *Figure S1B–D*, *S2F–G*, and *K–L* were analysed by two-sample *t*-test, and the data in Table S2 were analysed by χ2 test. One-way ANOVA or two-way ANOVA was conducted followed by Bonferroni *post hoc* test for comparisons among multiple groups. Specifically, the data in *Figures 1B, D–E*, *4J*, *5B* and *H*, Supplementary material online, *Figure S2B–E, J, S3A–D*, and *S4C* and *D* were analysed by one-way ANOVA, and those in *Figure 2B*–*D*, *F, H, J, L*, and Supplementary material online, *Figure S1E* and *F* were analysed by two-way ANOVA. *P*-values < 0.05 were considered statistically significant.

## 3. Results

### 3.1 miR-27b-3p expression is elevated in cardiac hypertrophy

To explore the role of miR-27-3p in cardiac remodelling, we first examined its expression by qPCR analysis. We examined the distribution of miR-27b-3p in different mouse tissues (heart, spleen, liver, kidney, brain, and lung) and observed that it is highly expressed in the heart (*Figure 1A*). Furthermore, the expression of miR-27b-3p increased with hypertrophy in a TAC-induced model (*Figure 1B*).

miR-27b-3p was also expressed in cardiomyocytes and cardiac fibroblasts (*Figure 1C*). Furthermore, miR-27b-3p expression increased in ISO-treated cardiomyocytes and cardiac fibroblasts relative to that in the vehicle control (*Figure 1D and E*).

### 3.2 Loss of miR-27b attenuates pathological cardiac remodelling induced by pressure overload

Next, we explored the functional role of miR-27b-3p in TAC-reduced pathological cardiac remodelling. In the TAC model, the aortic pressure gradient was higher in the TAC-operated group than in the sham-operated group (Supplementary material online, *Figure S1B*). miR-27b-3p expression was not detected in multiple tissues of KO mice (Supplementary material online, *Figure S1C*). We also evaluated the expression of miR-23-27-24 cluster members (including miR-27a, miR-23a, miR-23b, and miR-24) in the heart of WT and miR-27b KO mice. The expression of other miR-23-27-24 cluster members was not different between the two groups, indicating that miR-27b KO does not affect the expression of other members (Supplementary material online, *Figure S1D*). Echocardiography analysis of the cardiac function eight weeks after TAC showed shorter LVIDd, alongside higher LV EF%, and shortening rates (FS%) in miR-27b-null mice than in WT mice (*Figure 2B and C*). Echocardiography analysis of cardiac function eight weeks after TAC showed that the LV wall thickness during systole (LVPWs) and diastole (LVPWd) was significantly lower in miR-27b-null mice than in WT mice. The LV end-diastolic diameter (LVIDd)/LV end-systolic diameter (LVIDs), which expanded after TAC, was significantly reduced in miR-27b-null mice compared to that in WT mice (Supplementary material online, *Figure S1E*). Cardiac hypertrophy was also repressed in miR-27b-null mice, as shown by the heart weight/body weight ratio (*Figure 2D and E*). Cardiac size map showed that, after TAC, the heart was smaller in miR-27b-null than in WT mice (*Figure 2F*). We also found reduced fibrillary collagen deposition (Supplementary material online, *Figure S1F* and *Figure S2G–H*,) and Smad3 activation (a pro-fibrotic signalling) in miR-27b-null mice (Supplementary material online, *Figure S2A–C*). Moreover, the increased cross-sectional area of cardiomyocytes induced by TAC was significantly reduced in the absence of miR-27b (*Figure 2I and J*). Importantly, the expression of the inflammatory factors IL-β and MAC-2 (a marker of macrophages), which are pivotal during cardiac remodelling (15-16), was significantly down-regulated in miR-27b-null mice (Supplementary material online, *Figure S2D–E, K*–*L*).

To confirm improvement in cardiac remodelling in the absence of miR-27b-3p, RNA-sequencing was performed on cardiac tissues from WT and miR-27b-null mice post-TAC. We identified a total of 1228 differentially expressed genes (DEGs) between the two groups (Supplementary material online, *Supplemental Data*). Functional annotation of these genes using DAVID (https://david.ncifcrf.gov) showed that HCM-, TGF-β signalling-, and NF-κB signalling-related genes were significantly down-regulated in miR-27b-null heart (*Figure 2M and O*), which supported decreased pathological cardiac remodelling in miR-27b-null mice at the molecular level. Further, we found that miR-27b-3p was up-regulated in the left ventricular tissue of patients with HCM compared to that in healthy controls (*Figure 2P*).

### 3.3 Loss of miR-27b attenuates pathological cardiac remodelling induced by Ang II perfusion

Our previous studies demonstrated that AngII, a key component of the renin–angiotensin system (RAS), plays a key role in the pathogenesis of cardiac remodelling.^21^ We measured the levels of miR-27b-3p in WT and AngII-infused mice. An increased expression of miR-27b-3p was observed in AngII-infused mice (Supplementary material online, *Figure S2F*). To test the role of miR-27b-3p in Ang II-induced pathological cardiac remodelling, we infused miR-27b-null and WT mice with Ang II for 14 days. Similarly, loss of miR-27b significantly reduced heart size; miR-27b-null heart weight/body weight and heart weight/tibia length ratios were significantly decreased (*Figure 3A*). Echocardiography measurements showed that LV wall thickness was significantly lower in miR-27b-null than in WT mice after Ang II infusion (*Figure 3B–D*). The LVIDs and LVIDd of miR-27b-null mice were shorter than those of WT after Ang II infusion; the difference was not statistically significant, perhaps because cardiac hypertrophy was not obvious at 14 days of Ang II infusion (Supplementary material online, *Figure S2G*). In addition, miR-27b-null mice maintained LV function after Ang II perfusion and showed a significantly higher ventricular EF% than did WT mice (*Figure 3E*). Ang II significantly increased the myocardial cross-sectional area in the WT group, an effect which was substantially abolished by loss of miR-27b (*Figure 3F*). Additionally, cardiac fibrosis was suppressed, and IL-1β expression was significantly lower in miR-27b-null mice after Ang II perfusion (*Figure 3G and H*).

### 3.4 *FGF1* serves as a target gene of miR-27b-3p

Next, we performed RNA-sequencing to profile the transcriptomes of miR-27b-null and WT hearts eight weeks after TAC, and found that 607 genes were up-regulated in the absence of miR-27b. Ninety-two candidates were obtained by intersecting up-regulated genes with miR-27b target genes using prediction systems (http://www.targetscan.org and http://mirdb.org); the top 16 genes were chosen as candidate targets for further validation (*Figure 4A*, Supplementary material online, *Figure S2H*). We examined whether these candidate genes could be inhibited by miR-27b-3p mimic in H9c2 cells. As detected by qPCR, three targets (*FGF1, PPAR-γ*, and *Dram2*) were significantly inhibited by miR-27b-3p mimic compared to the NC mimic (*Figure 4B*). miR-27b-3p has only one binding site in the 3'-UTR of *PPAR-γ* (a known target of miR-27b)^22^ and *Dram2* (an oncogenic regulator) (Supplementary material online, *Figure S2I*), but three in the 3'-UTR of *FGF1* (*Figure 4C*). *FGF1*, a member of the FGF family, is crucial for regulation of glucose and fat metabolism.^23,24^ We detected the FGF1 protein levels in multiple organs and found that it is highly expressed in the heart (Supplementary material online, *Figure S2J*). Therefore, we speculated that *FGF1* might be a target of miR-27b-3p involved in cardiac remodelling.

The expression of miR-27b-3p was seven times higher in H9c2 cells transfected with miR-27b mimic than in NC mimic transfected cells. As the miR inhibitor was a single-stranded antisense RNA against miRNAs with 2’-O-Me modification, it could not change the expression of miR. Alternatively, the protein expression of PPAR-γ, a known target of miR-27b, increased by 50% after miR-27b inhibitor transfection (Supplementary material online, *Figure S2K–L*). Luciferase reporter assay revealed that miR-27b-3p can repress *FGF1* by binding its 3'-UTR. When the three binding sites of miR-27b-3p were mutated, the repression was lost (*Figure 4D*), demonstrating that miR-27b-3p directly targets *FGF1*. Further, qPCR analysis confirmed that miR-27b-3p mimic treatment inhibits FGF1 expression in isolated cardiomyocytes (*Figure 4E*). Moreover, FGF1 protein expression increased in cardiomyocytes treated with miR-27b-3p inhibitor (*Figure 4F*). Consistent with *in vitro* observations, the expression of FGF1 mRNA and protein was significantly higher in miR-27b-null mice than in WT mice (Supplementary material online, *Figure S4G* and *H*). Similarly, ELISA showed that the concentration of FGF1 increased in the serum and heart of miR-27b-null mice (*Figure 4I*). Moreover, we found that FGF1 expression in miR-27b KO mice was significantly increased in the TAC model compared to that in WT mice (Supplementary material online, *Figure S4J*). These results indicate that *FGF1* serves as a target gene of miR-27b-3p.

### 3.5 FGF1 enhances mitochondrial oxidative function

Next, we investigated whether FGF1 is involved in cardiomyocyte hypertrophy. To analyse morphological changes, we treated cardiomyocytes with rFGF1, miR-27b-3p inhibitor, or miR-27b-3p mimic for 48 h, then immunostained for α-actinin. PE-induced hypertrophy was decreased in cardiomyocytes treated with either rFGF1 or miR-27b inhibitor when comparing with that in cardiomyocytes treated with vehicle or NC inhibitor (*Figure 5A and B*). Additionally, in response to the miR-27b-3p stimulus, cardiomyocyte area, atrial natriuretic peptide (ANP), and BNP mRNA levels were decreased in rFGF1-treated cardiomyocytes compared with those in vehicle-treated cardiomyocytes (Supplementary material online, *Figure S3A–C*). Similarly, the protein levels of ANP and BNP were decreased in rFGF1-treated cardiomyocytes compared with those in vehicle-treated cardiomyocytes (Supplementary material online, *Figure S3D*). Functional annotation of the cardiac DEGs between miR-27b-null and WT mice post-TAC using DAVID revealed forward signalling in multiple metabolic processes, including oxidative phosphorylation, tricarboxylic acid cycle, and fatty acid oxidation (*Figure 5C*, Supplementary material online, *Figure S4A*). Therefore, we hypothesized that FGF1 up-regulation might be responsible for the enhanced mitochondrial function. We then determined the effect of rFGF1 on mitochondrial respiratory capacity by measuring OCR, a strong indicator of mitochondrial function, with seahorse XF. Compared with vehicle, rFGF1 treatment significantly up-regulated the basal respiration and maximum respiratory capacity in cardiomyocytes (*Figure 5D and F*). Dysfunction of energy metabolism is an important part of cardiac pathological remodelling.^25,26^ As peroxisome proliferator-activated receptor γ coactivator-1α/β (PGC1-α/PGC1β) are important regulators of mitochondrial function,^27,28^ we tested whether rFGF1 treatment up-regulated their expression in the heart. PGC1-α/β protein levels were higher in the heart from rFGF1 treatment than that of vehicle treatment (Supplementary material online, *Figure S5G*). Next, we knocked down FGFR1 to investigate whether FGF1 enhances the expression of PGC1-α/β via its receptor. The results showed that compared to vehicle control, rFGF1 treatment significantly increased the expression of PGC1-α/β in H9c2 cells, but the effect was abolished when FGFR1 was knockdown (Supplementary material online, *Figure S5H*). These findings indicated that FGF1-FGF1R signalling enhances mitochondrial oxidative function via up-regulating PGC1-α/β.

### 3.6 FGF1 attenuates the pathological cardiac remodelling induced by TAC and Ang II

As FGF1 improved mitochondrial oxidative phosphorylation, we explored the role of FGF1 in pathological cardiac remodelling using two independent cardiac hypertrophy models, TAC and Ang II. As shown in Supplementary material online, *Figure S4B*, WT mice (8–10 weeks old) received intraperitoneal injection of rFGF1 at a dose of 0.5 mg/kg or saline every other day from the day of TAC operation or Ang II infusion, until the end of the experiments. TAC induced a significant increase in the size of WT mouse hearts, but this was substantially suppressed by rFGF1 treatment (*Figure 6A*). Moreover, heart weight/body weight and heart weight/tibia length ratios significantly decreased in rFGF1-treated mice (*Figure 6B and C*). Echocardiography showed that the LV wall thickness and LV mass were significantly lower in rFGF1- than in the vehicle-treated group (*Figure 6D and E*). EF% and FS% were also significantly higher following rFGF1 treatment (*Figure 6E and F*), demonstrating that rFGF1 can attenuate the decrease in cardiac contractility caused by TAC. The cross-sectional area of cardiomyocytes was also significantly reduced upon rFGF1 treatment compared to that in the vehicle treatment (*Figure 6H*). Finally, the interstitial and perivascular fibrosis area and the expression of p-Smad3 also significantly decreased in rFGF1-treated mice (*Figure 6I*, Supplementary material online, *Figure S4C*).

We also explored the role of FGF1 in pathological cardiac remodelling through Ang II perfusion. rFGF1 treatment substantially suppressed the increase in heart size caused by Ang II (*Figure 6J*). The heart weight/body weight and heart weight/tibia length ratios significantly decreased in the rFGF1 group (*Figure 6K and L*). Echocardiographic analysis showed that in rFGF1-treated mice, the LV wall thickness, LV mass/BW, and LVPWs were significantly lower, while FS% was significantly higher than those in the vehicle-treated mice (*Figure 6M–P*). Finally, rFGF1 treatment significantly reduced the myocardial cell cross-sectional area (*Figure 6Q*) and cardiac fibrosis (*Figure 6R*). Collectively, our data indicated that rFGF1 treatment could reduce cardiac hypertrophy, fibrosis, and contractile dysfunction under pressure overload or Ang II infusion.

## 4. Discussion

In this study, we demonstrated that pathological cardiac remodelling, induced by pressure overload or Ang II infusion, was attenuated in miR-27b-null mice. miR-27b-3p inhibited the expression of FGF1, which promoted mitochondrial oxidative phosphorylation via PGC1α/PGC1β. Finally, rFGF1 treatment attenuated the pathological cardiac remodelling in both the TAC and the Ang II infusion models.

Different mechanisms by which miRNAs regulate HF and cardiac remodelling have been described. It would thus be ideal to test mir-27b-3p expression in heart samples from patients with severe aortic stenosis and long-standing hypertension. However, it is difficult to obtain cardiac samples from these patients. Moreover, although HCM is a genetic cardiomyocyte-intrinsic disease, there are common pathological changes (such as cardiomyocyte hypertrophy and fibrosis) and common activating signal pathways (such as TGF-β/SMAD3) between HCM and severe aortic stenosis or long-standing hypertension.^29,30^ Thus, we tested whether mir-27b-3p was up-regulated in hypertrophic heart tissue from patients with HCM. Here, we found that miR-27b-3p expression was elevated in the heart of cardiac hypertrophy patients and in TAC-operated mice. Considering that miRNAs are small (21–22 nucleotides) non-coding RNAs that can diffuse into serum, our results might explain the previous finding that circulating miR-27b levels are higher in patients with hypertrophic cardiomyopathy than in healthy controls.^7^ Previous studies proved that cardiomyocyte-specific overexpression of miR-27b induces cardiac hypertrophy and dysfunction in mice.^15^ However, different studies demonstrated that overexpression of specific miRNAs cannot fully recapitulate their endogenous role. For example, miR-22 had been linked to cancer and considered a tumour suppressor.^31^ In contrast, miR-22 overexpression induces myelodysplastic syndrome and bone marrow malignancies in mice.^32,33^ These contradictory data suggest that overexpression of miRNAs does not reveal the actual physiological/pathological role of endogenous miRNAs. In the present study, we proved from multiple angles that miR-27b inhibition attenuates pathological cardiac remodelling, as follows: (i) pathological cardiac remodelling was attenuated by systemic loss of miR-27b function in two established cardiac hypertrophy mouse models; (ii) multi-dimension data demonstrated the protective role of miR-27b loss in cardiac remodelling, including echocardiographic analysis showing improvement of cardiac systolic/diastolic function; (iii) pathological examination showed decrease of collagen deposition and reduction of cardiomyocyte hypertrophy and inflammatory factors; and (iv) unbiased RNA-sequencing analysis revealed that miR-27b-null significantly repressed the pro-fibrotic (i.e. TGF-β), hypertrophic (i.e. HCM), and pro-inflammatory (i.e. NF-κB) pathways. These robust findings demonstrated that inhibition of miR-27b plays a protective role in pathological cardiac remodelling.

In search of genes regulated by miR-27b-3p, we found that FGF1 was significantly up-regulated in miR-27b-null mice. In comparison with that in vehicle-treated mice, FGF1 protein expression in miR-27b KO mice was significantly increased. FGF1 is an autocrine/paracrine factor, and recombinant human FGF1 has been clinically used to facilitate wound/burn repair and ulcer regeneration for decades.^34,35^ Recently, FGF1 was shown to be crucial for the management of nutrient stress, glycaemic control, and insulin sensitivity.^24,36,37^ Jonker *et al*.^38^ discovered an unexpected metabolic role for FGF1 as a critical transducer of PPAR-γ signalling, coupling nutrient storage to adaptive remodelling of adipose tissue. FGF1 mRNA is highly expressed in multiple tissues,^39^ and we found that FGF1 protein was enriched in the heart and kidney. Cardiac overexpression of FGF1 can promote myocardial cell survival and reduce infarct size under myocardial infarction.^40^ Although FGF1 activates MAPKs, which promote mitosis and cause cell division and proliferation,^24,41^ to our knowledge, we demonstrated for the first time that rFGF1 inhibits pathological cardiac remodelling in two established animal models of cardiac hypertrophy. Herein, we found that FGF1 up-regulated PGC-1α/β and increased mitochondrial oxidative phosphorylation. This novel function is distinct from its previously known role in activation of PLCγ and MAPK/AKT pathways.^42,43^ The transcriptional coactivators PGC-1α/β are major regulators of oxidative phosphorylation and fatty acid oxidation-related gene expression, and play a key role in regulating pressure overload-induced cardiac hypertrophy and HF.^28,44,45^ Treatment with the PGC-1α stimulator, rosiglitazone, enhances mitochondrial oxidative phosphorylation, and maintaining physiological expression of PGC-1α/β can sustain systolic function and attenuate the remodelling of heart disease caused by pressure overload.^46,47^ The benefit gained by the abrogation of miR-27b-3p expression was greater than that of FGF1 supplementation alone. *FGF1* is one of the target genes of miR-27b-3p. *PPAR-γ* and *UCP1* are also the target genes of miR-27b-3p.^15,17^ Both *PPAR-γ* and *UCP1* play antagonistic roles in cardiac hypertrophy. *PPAR-γ* deficiency in mice aggravates stress-induced cardiac hypertrophy and dysfunction.^48^*UCP1* deficiency exacerbates myocardial injury, fibrosis, and adverse remodelling, as well as mortality induced by isoproterenol infusion in mice.^17,49^ Unlike the anti-hypertrophic effect of FGF1 involving the improvement of mitochondrial OXPHOS, miR-27b deficiency suppressed cardiac hypertrophy through up-regulation of multiple target genes, such as *PPAR-γ, FGF1*, and *UCP1*. The involvement of multiple pathways may explain the greater benefit of abrogating miR-27b-3p expression relative to that obtained by FGF1 replacement. The expression of miR-27a was enhanced in the heart following TAC (Supplementary material online, *Figure S4D*). Our study and a previous study^50^ showed that *FGF1* is a target of both miR-27a and miR-27b-3p. Further research is warranted to fully elucidate the specific effect of miR-27a on cardiac hypertrophy.

In summary, we demonstrated that inhibition of miR-27b-3p plays a protective role in the development of pathological cardiac remodelling. We identified that FGF1, as a target gene of miR-27b-3p, enhances mitochondrial OXPHOS and PGC1α/β. Administration of rFGF1 protects against pathological cardiac remodelling. Our findings suggest that miR-27b-3p and *FGF1* may be potential therapeutic targets to treat conditions characterized by pathological cardiac remodelling.

## Supplementary material

Supplementary material is available at *Cardiovascular Research* online.

Translational perspective

MicroRNAs (miRNAs) are involved in post-transcriptional regulation of genes in cardiac physiology and pathology. However, the mechanisms underlying miRNA-mediated regulation of pathological cardiac remodelling remain to be studied. We show for the first time that miR-27b deletion attenuates cardiac hypertrophy, fibrosis, and inflammation and that rFGF1 administration inhibits cardiac hypertrophy and fibrosis in TAC- or Ang II-induced models, and enhances OXPHOS by activating PGC1α/β. Our findings suggest that miR-27b-3p and *FGF1* may be potential therapeutic targets to treat conditions characterized by pathological cardiac remodelling.

## Supplementary Material

cvab248_Supplementary_Data

## Data Availability

The mRNA sequencing data have been deposited in the Gene Expression Omnibus under accession number GSE158191. The data underlying this article will be shared on reasonable request to the corresponding author.
