# Supplementary material for: MicroRNA-27b-3p down-regulates FGF1 and aggravates pathological cardiac remodelling
Source: Cardiovasc Res. 2021 Aug 6;118(9):2139–51. doi: 10.1093/cvr/cvab248 (PMC9302889; doi:10.1093/cvr/cvab248)
Supplement: cvab248_Supplementary_Data [file cvab248_supplementary_data.pdf]

MicroRNA-27b-3p downregulates *FGF1* and aggravates pathological cardiac remodelling

Guoqi Li[#], Yihui Shao[#], Hong-Chang Guo, Ying Zhi, Bokang Qiao, Ke Ma, Yong-Qiang Lai*, Jie Du *, Yulin Li *

Beijing Anzhen Hospital, Capital Medical University; The Key Laboratory of Remodelling-Related Cardiovascular Diseases, Ministry of Education; Beijing Institute of Heart Lung and Blood Vessel Diseases, Beijing 100029, China

# equal contribution

*Corresponding author: Yong-Qiang Lai yongqianglai@yahoo.com or Jie Du, jiedu@ccmu.edu.cn or Yulin Li, lyllyl_1111@163.com

(Tel: 86-10-64456030; fax: 86-10-64456094).

S1:Loss of miR-27b attenuates pathological cardiac remodelling in mice undergone pressure overload.

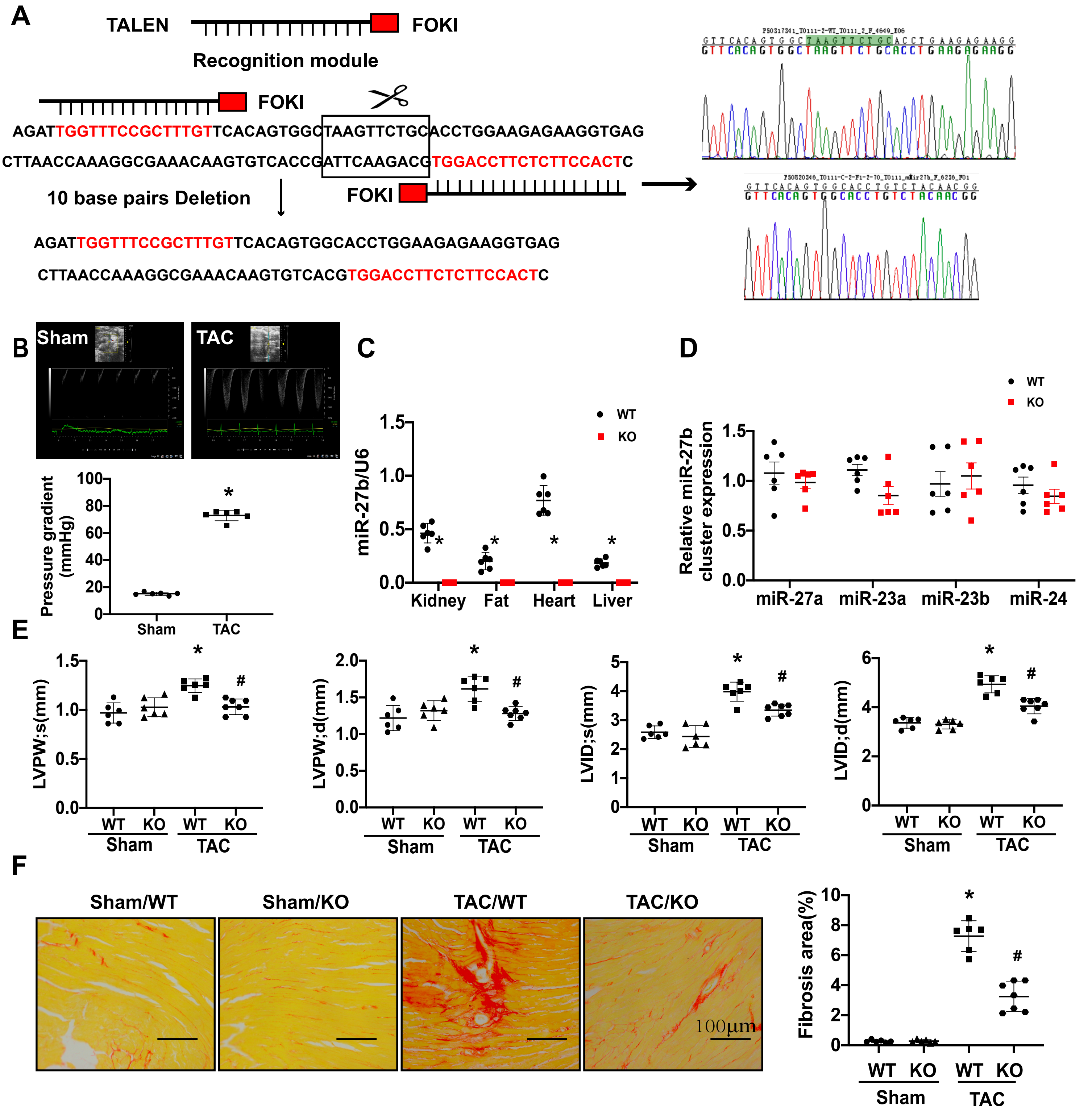

A, The schematic diagram of generating transgenic mice (miR-27b-null),The miR-27b-null mice were generated using TALEN technology, with 10bp deletion in the 3' end. B, PW Doppler images of the aorta from sham- or TAC-operated mice are shown. The peak aortic velocity obtained from PW Doppler imaging was used to calculate the pressure gradient according to the modified Bernoulli equation. These data confirm the success of the TAC surgery, which generated a pressure gradient of ~70 mmHg. *P < 0.05 vs. Sham. (n = 6). C, miR-27b was expressed in multiple tissues of wildtype and knockout mice (n = 6). D, qPCR shows expression of miR-27b-3p cluster (miR-27a，miR-23a, miR-23b，miR-24) in heart tissue of wild type and knockout mice (n = 6). E, Measurements of the LV wall thickness during systole (LVPWs) and diastole (LVPWd) and the LV internal diameters during systole (LVIDs) and diastole (LVIDd) in wild-type and KO mice after TAC surgery or sham operation. (WT n = 6, KO, n = 7). F, Representative images of heart sections from wild-type and KO mice were stained with Sirius Red's staining after sham or TAC operation. Quantification of the fibrosis area in wild-type and KO mice after TAC or sham operation (WT n = 6, KO, n = 7). All data are shown as mean ± SD.*P < 0.05 compared to sham (B) or WT (C, D) or sham/WT (E, F); #P<0.05 compared to WT/TAC. For two groups, data were compared by 2-sample t test; for more than two groups, data were compared by two-way ANOVA with Bonferroni post-hoc test.

S2:identified the candidate target genes of miR-27b-3p

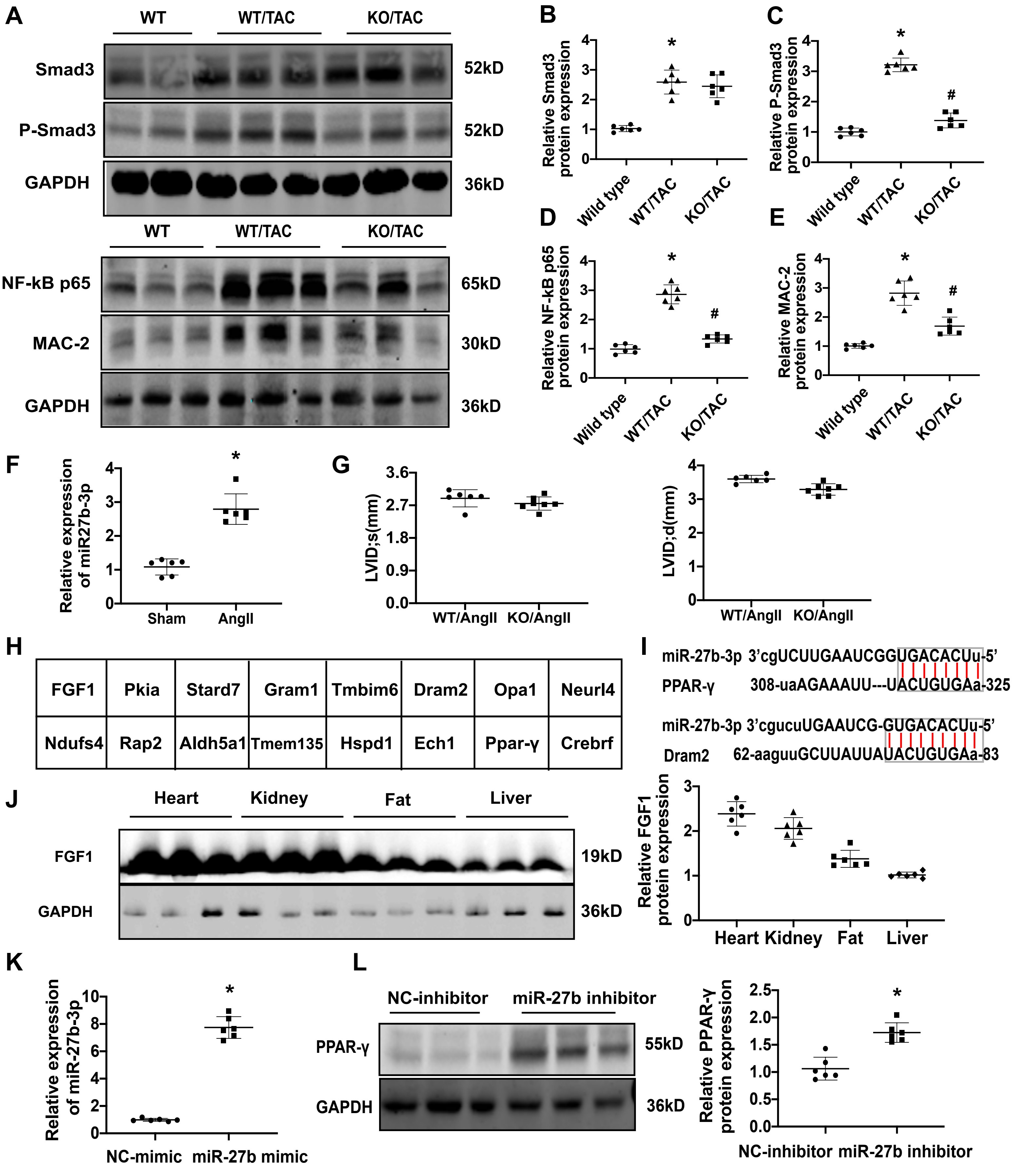

A, Western blot image of protein expression in whole heart of wildtype and miR-27b-3p-null mice after TAC. B-E, Quantification of protein expression of Smad3, P-Smad3, NF-κB p65, and MAC-2 (n = 6), normalised to GAPDH. F, qPCR shows expression of miR-27b-3p in heart tissue after sham or AngII infusion operation. (n = 6). G, Measurements of the LV internal diameters during systole (LVIDs) and diastole (LVIDd) in wild-type and miR-27b-3p-null mice after AngII infusion operation. (WT n = 6, KO, n = 7). H, Sixteen genes selected as candidate target genes of miR-27b-3p. I, Predicted miR-27b-3p binding sites on PPAR-γ and Dram2. J, Western blot analysis shows the expression of FGF1 in different tissues (heart, kidney, fat, liver) (n = 6). K-L, qPCR shows expression of miR-27b-3p in H9c2 cells treated with NC-mimic or miR-27b-3p mimic, western blot analysis shows the protein expression of PPAR-γ in H9c2 cells treated with NC-inhibitor or miR-27b-3p inhibitor (n = 6). All data are shown as mean ± SD. *P < 0.05 compared to WT (B, C, D, E) or sham (F) or NC-mimic (K), NC-inhibitor (L); #P<0.05 compared to WT/TAC. For two groups, data were compared by 2-sample t test; for more than two groups, data were compared by one-way ANOVA with Bonferroni post-hoc test.

S3:rFGF1 inhibits isolated neonatal mouse cardiomyocytes (NCMs)hypertrophy was induced by miR-27b-3p mimic

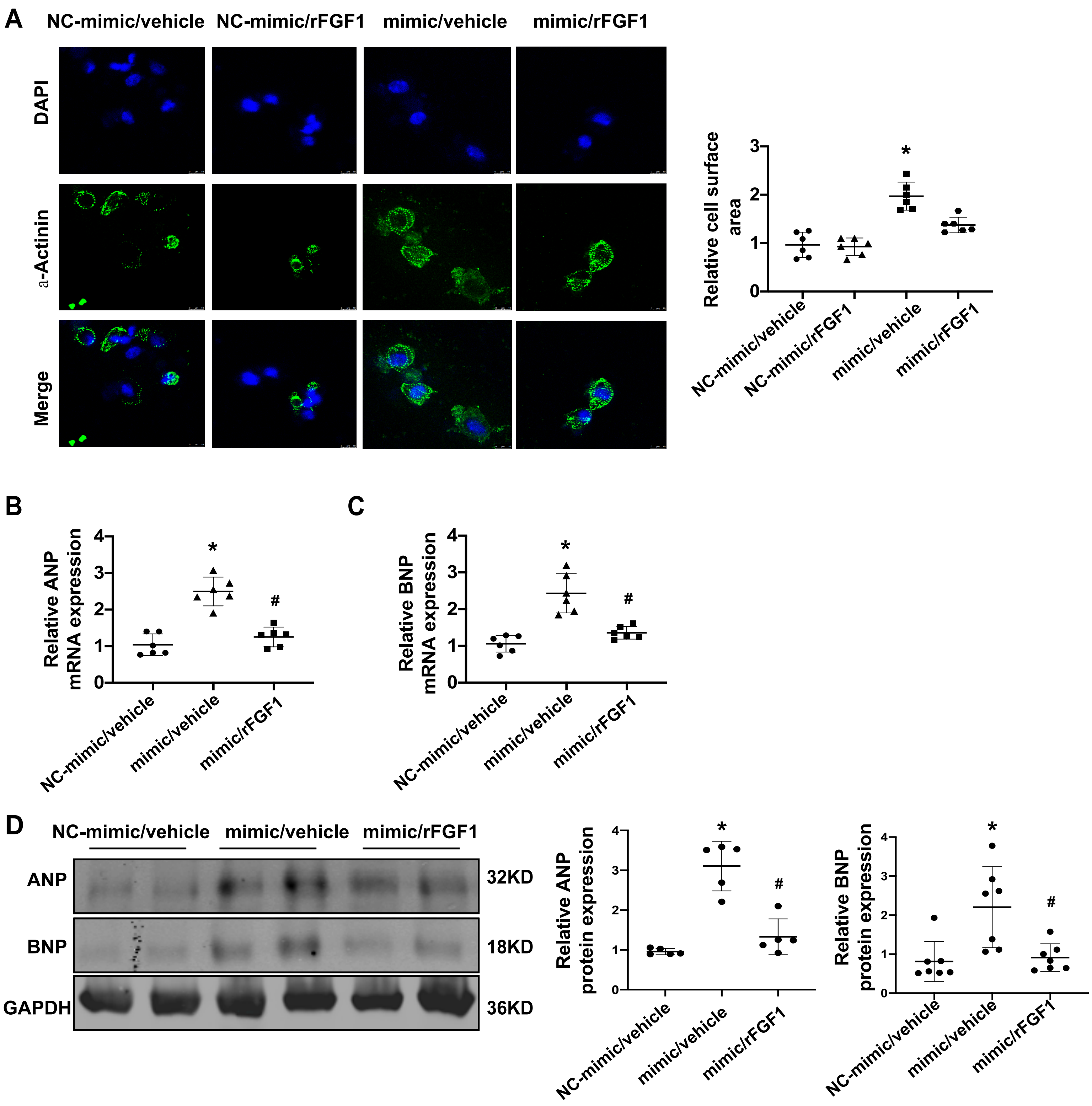

A-C, Isolated neonatal mouse cardiomyocytes (NCMs) were administered rFGF1 or miR-27b-3p mimic, NC-mimic or vehicle as control, respectively. Cardiomyocytes were stained for α-actinin (green), and DAPI staining was used to visualise the nuclei (blue). Scale bar, 15 μm. ANP and BNP transcript levels were quantitated using real-time PCR. D, Western blot analysis shows the protein expression of BNP, ANP in rFGF1 treated primary cardiomyocytes with miR-27b-3p mimic or NC-mimic, GAPDH as a loading control (n = 6). *P < 0.05 vs NC-mimic/vehicle. #P < 0.05 vs mimic/vehicle. Data were compared by one-way ANOVA with Bonferroni post-hoc test.

S4:FGF1 attenuates the pathological cardiac remodelling

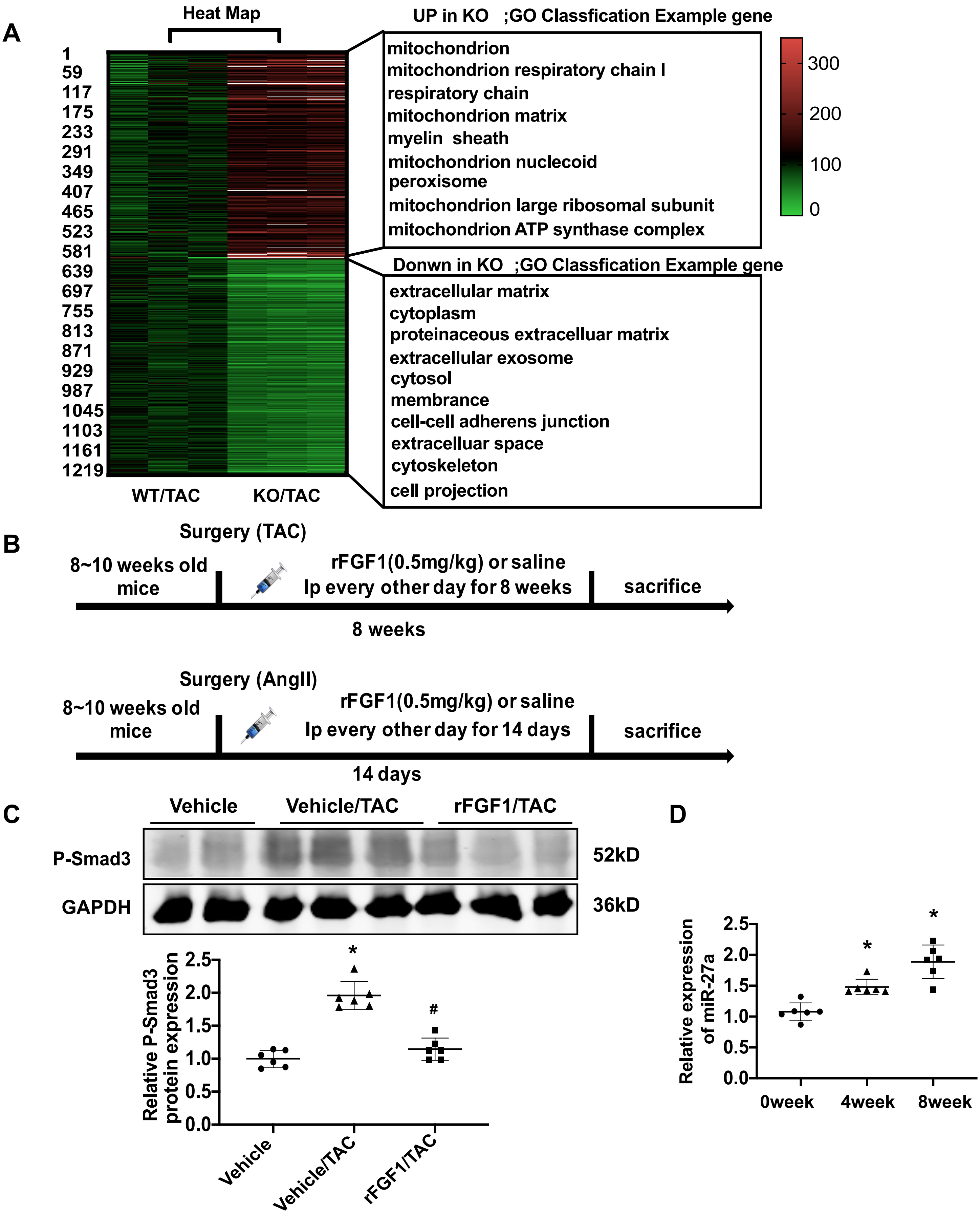

A, Heatmaps of the relative expression of the differentially expressed genes identified in RNA-sequencing of whole heart after pressure overload. B, The schematic diagram of beginning, duration and end time of the rFGF1 treatment. C, Western blot analysis shows the expression of P-Smad3 in vehicle- or rFGF1-treated mice undergone pressure overload (n = 6). D, qPCR shows expression of miR-27a in heart tissue after TAC 0, 4, and 8 weeks of mice (n = 6). *P < 0.05 compared to vehicle (C), or 0 week (D). #P < 0.05 compared to vehicle/TAC (C). Data were compared by one-way ANOVA with Bonferroni post-hoc test.

SUPPLEMENTAL MATERIAL

Original Western Blots used for Figure 4F

Western blot analysis of FGF1 protein expression in NC-inhibitor or miR-27b-3p inhibitor treated cardiomyocytes, GAPDH was used as a loading control.

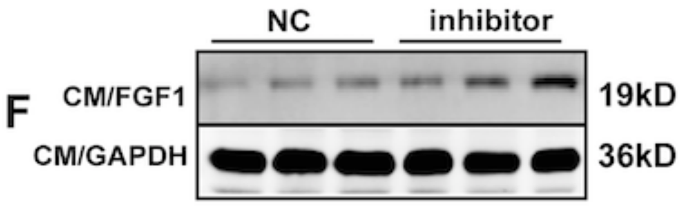

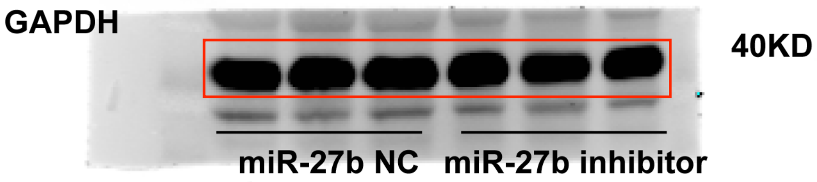

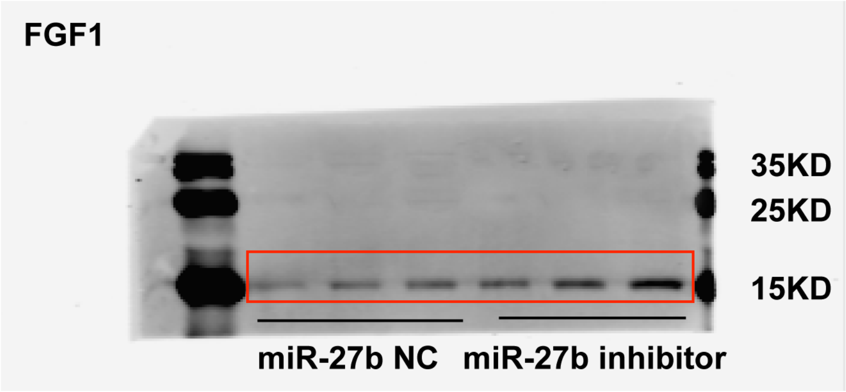

Original Western Blots used for Figure 4H

Western blot analysis of FGF1 protein expression in whole heart of wildtype or miR-27b-3p-null mice, GAPDH was used as a loading control.

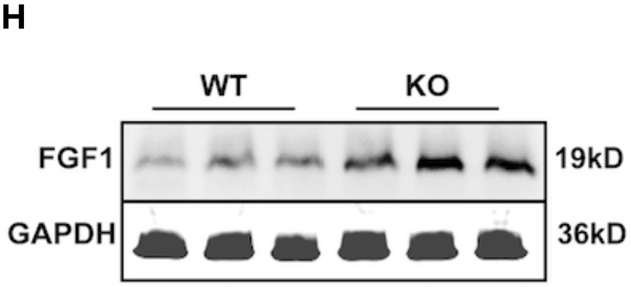

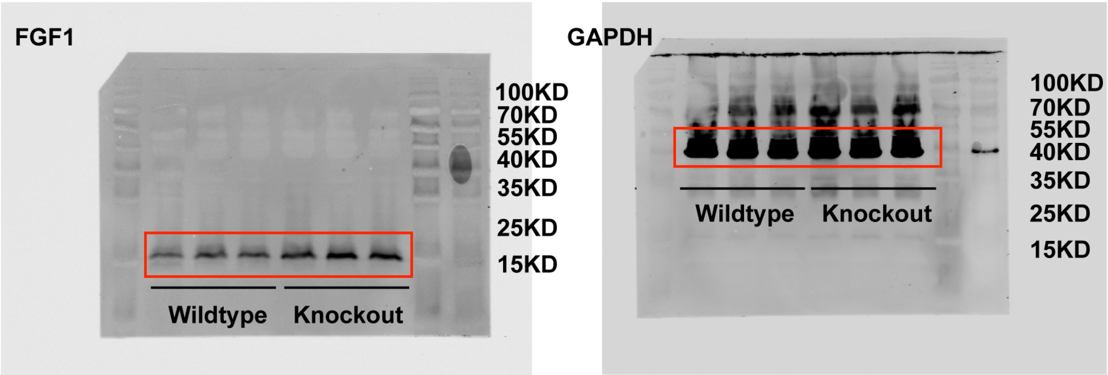

Original Western Blots used for Figure 4J

Western blot analysis of FGF1 protein expression in whole heart of wildtype and mir27b-3p-null mice after TAC, GAPDH was used as a loading control.

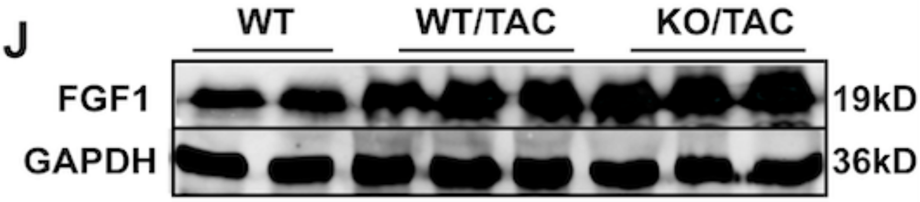

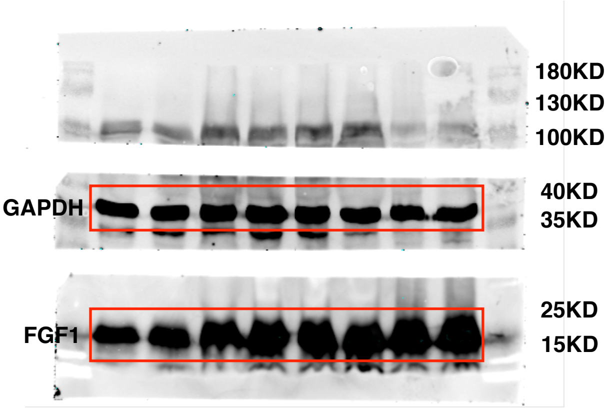

Original Western Blots used for Figure5G

Immunoblot for PGC-1α/PGC-1β in the heart from vehicle or rFGF1-treated 8 weeks in mice after TAC.

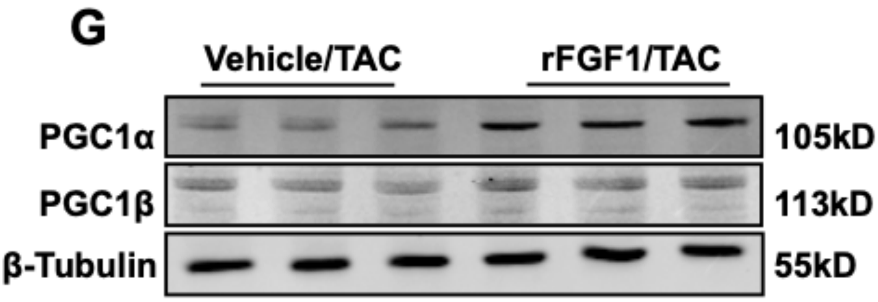

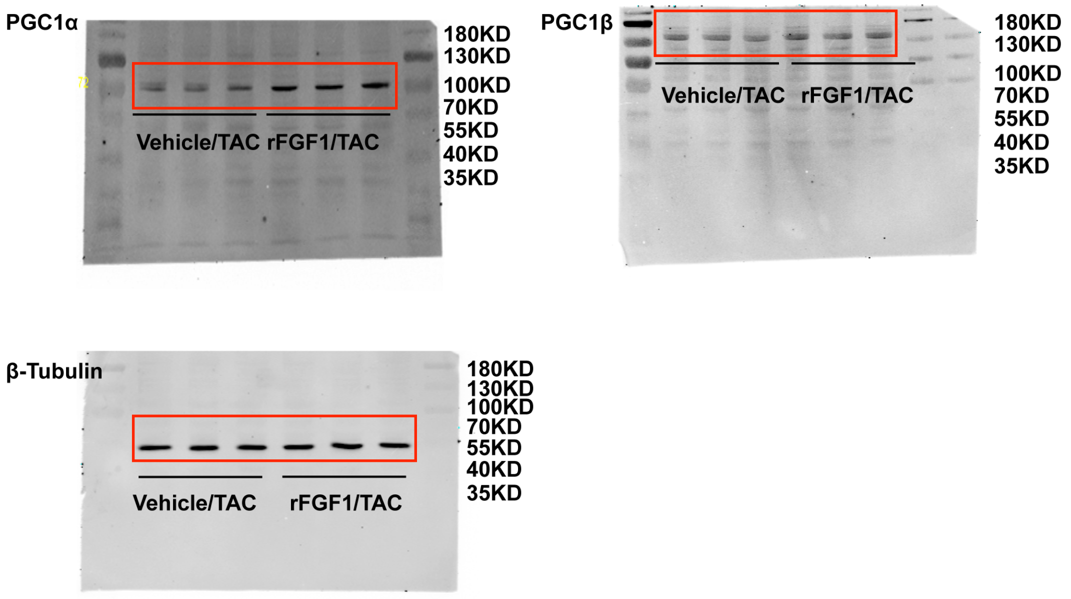

Original Western Blots used for Figure5H

Immunoblot for PGC-1α/PGC-1β in H9c2 cells treated with vehicle or rFGF1, rFGF1/siFGFR1 for 24 hours.

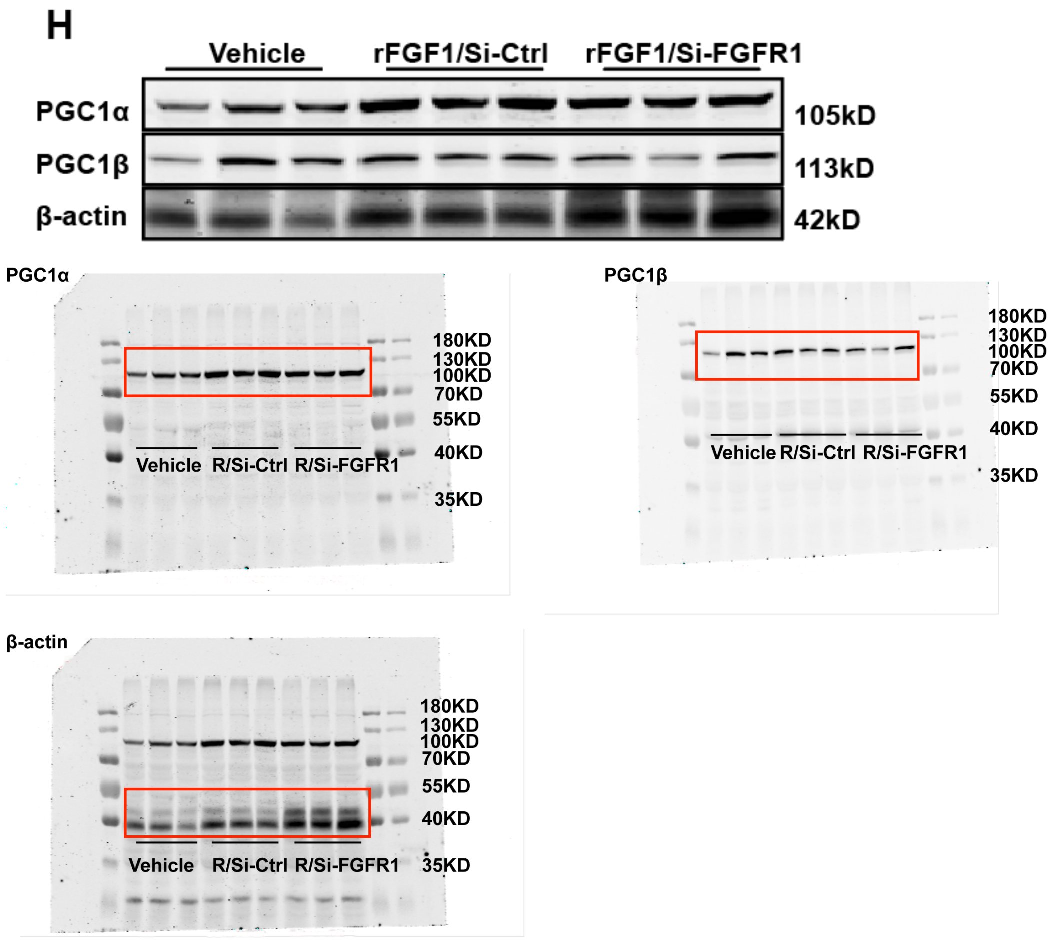

Original Western Blots used for Supplemental Figure 2J

Western blot analysis shows the protein expression of FGF1 in different tissues (heart, kidney, fat, liver).

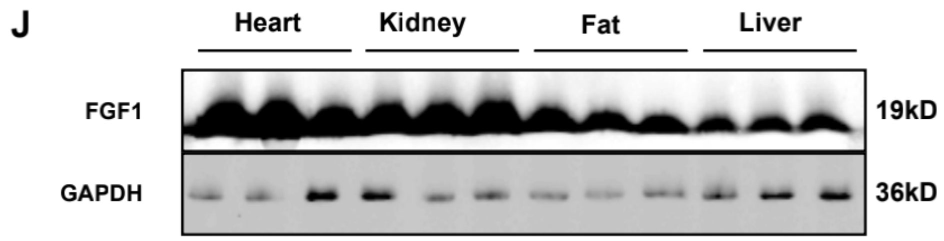

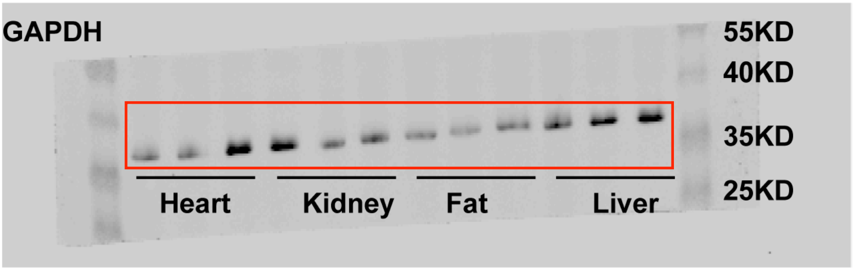

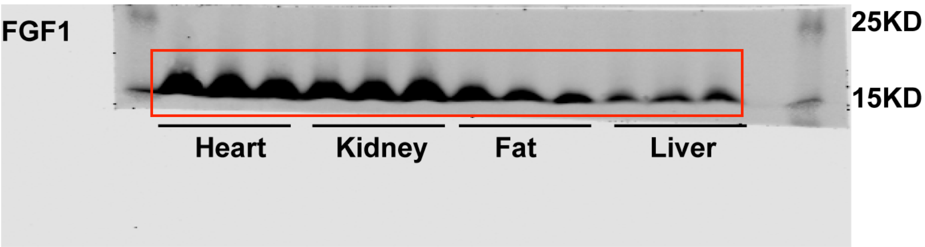

Original Western Blots used for Supplemental Figure 2A

Western blot shows the protein expression of Smad3 and p-Sma3, NF-kB, MAC-2 protein expression in whole heart of wildtype and miR-27b-3p-null mice after TAC.

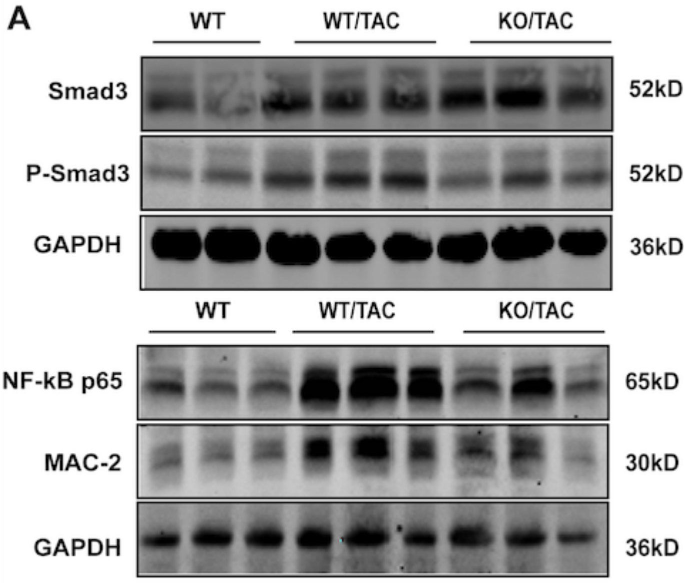

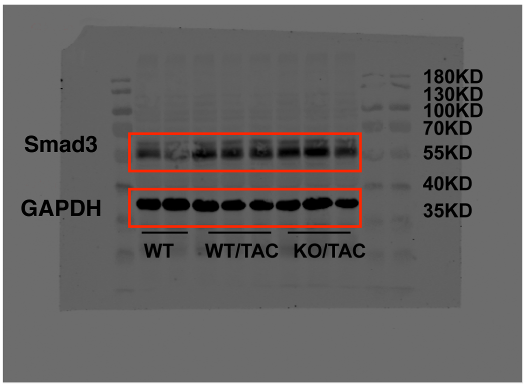

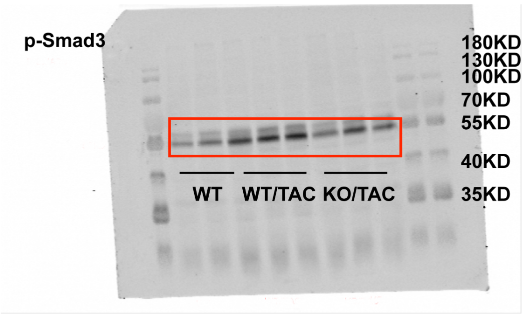

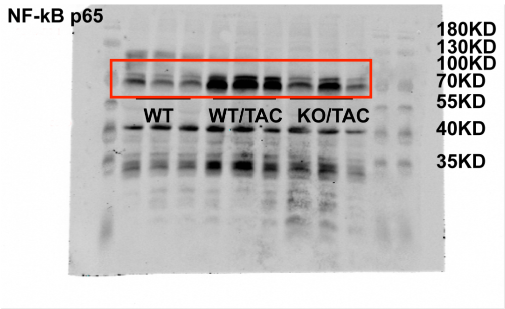

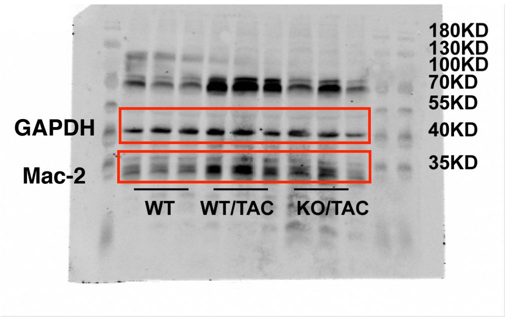

Original Western Blots used for Supplemental Figure 2L

Western blot analysis shows the protein expression of PPAR-γ in H9c2 cells treated with NC-inhibitor or miR-27b-3p inhibitor.

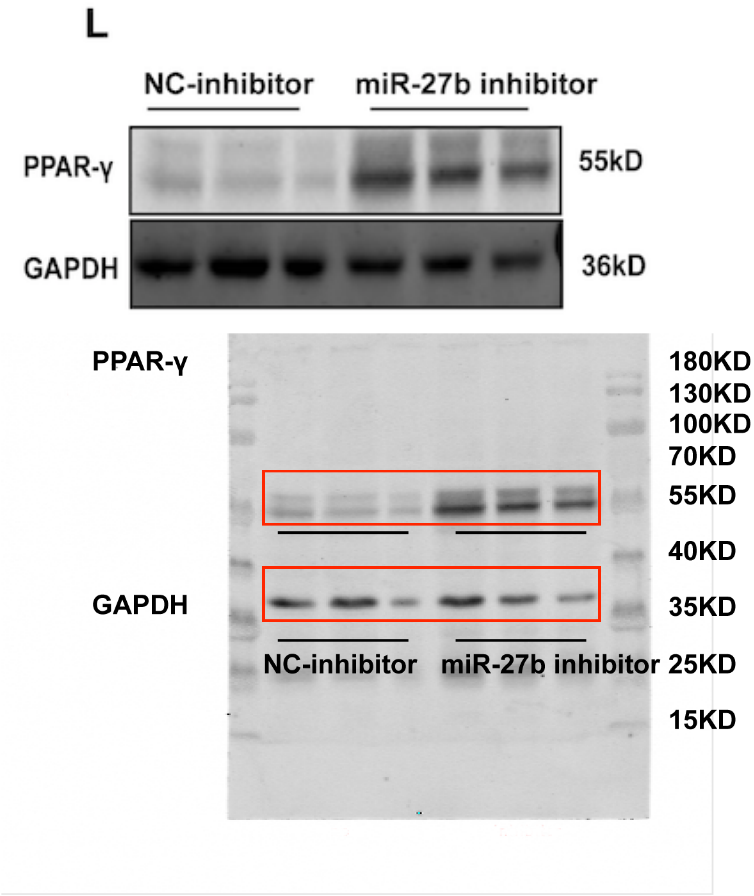

Original Western Blots used for Supplemental Figure 3D

Western blot analysis shows the protein expression of BNP, ANP in rFGF1 treated primary cardiomyocytes with miR-27b-3p mimic or NC-mimic.

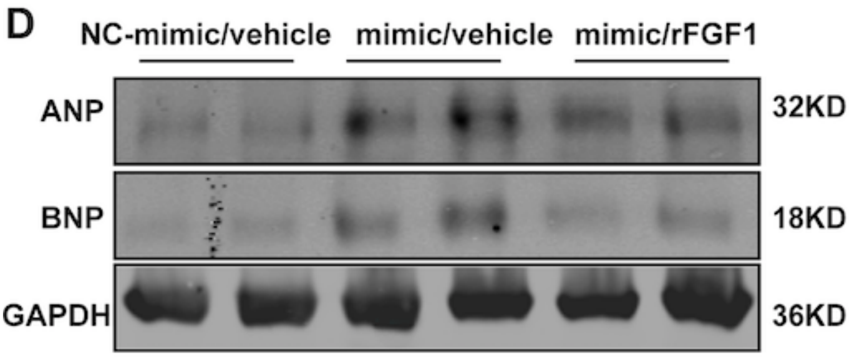

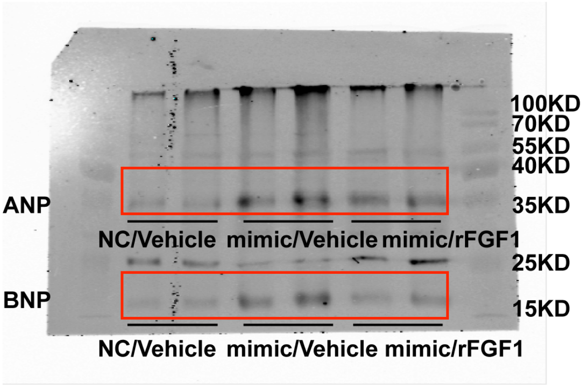

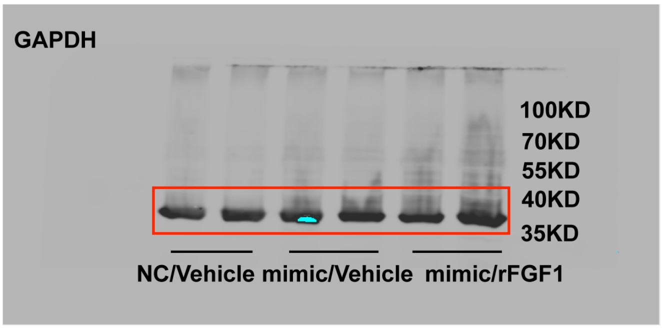

Original Western Blots used for Supplemental Figure 4C

Western blot analysis shows the protein expression of p-Smad3 in the heart from vehicle or rFGF1-treated 8 weeks in mice after TAC.

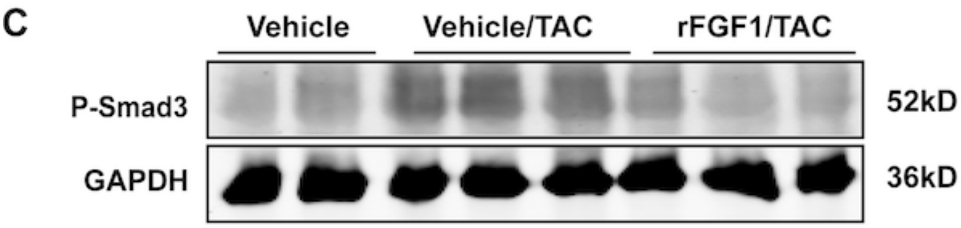

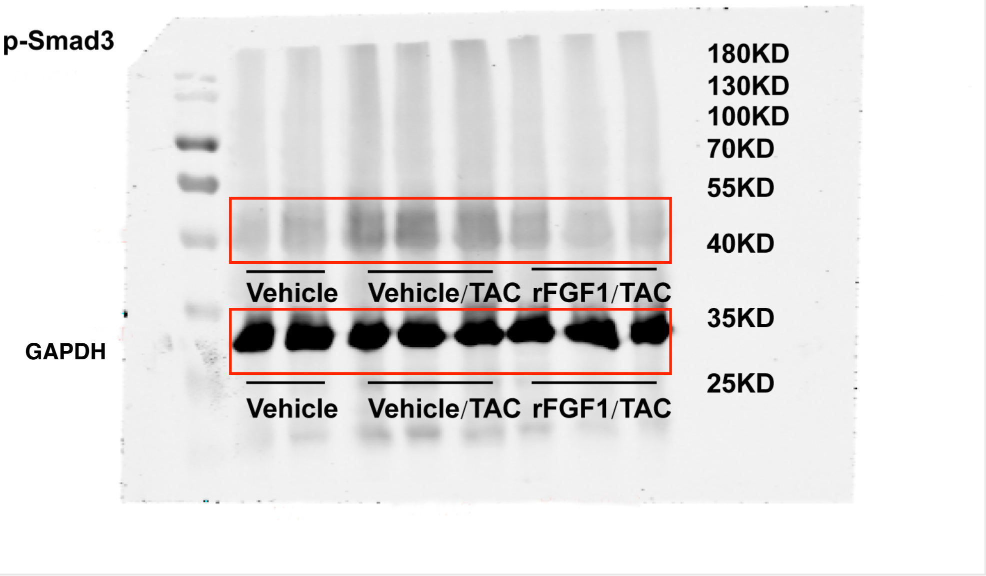

Table S1 The List Primer sequence of mRNA

Number	Primer	Primer sequence (5’ to 3’)
1	mMir27b-F	CGCCTTGTGGCTCTTTGGAAA
2	mMir27b-R	TGACAACACAGCCTTGAGGCAG
3	IL-1b-F	TTACAGTGGCAATGAGGATGAC
4	IL-1b-R	GTCGGAGATTCGTAGCTGGAT
5	Fgf1-F	CCCTGACCGAGAGGTTCAAC
6	Fgf1-R	GTCCCTTGTCCCATCCACG
7	Grm1-F	TGGAACAGAGCATTGAGTTCATC
8	Grm1-R	CAATAGGCTTCTTAGTCCTGCC
9	Ndufs4-F	CTGCCGTTTCCGTCTGTAGAG
10	Ndufs4-R	TGTTATTGCGAGCAGGAACAAA
11	Pkia-F	AGACAGAAGGTGAAGATGATGG
12	Pkia-R	AGCAATGCCAGGAGATTCG
13	Ech1-F	GCTACCGCGATGACAGTTTC
14	Ech1-R	TCAGAGATCGAAGGCTGATGTT
15	Dram2-F	GCTGTCCTTGCCTTTAGTATGG
16	Dram2-R	AGATAACCAACAGTAGTCGGACC
17	Eepd1-F	GGCTGCCATCGCTCTATCC
18	Eepd1-R	GGCTGCCATCGCTCTATCC
19	Tmem135-F	TCCCCTCTGCGTTTAGGCA
20	Tmem135-R	GGGCATGGTGGATTCTGTGT
21	Opa1-F	TGGAAAATGGTTCGAGAGTCAG
22	Opa1-R	CATTCCGTCTCTAGGTTAAAGCG
23	Crebrf-F	AGCGTAAGCGGAATGGACC
24	Crebrf-R	CAGGACATCTGTGAAAGTCTCC
25	Stard7-F	ACCAGTACCGAGTTTTTGGAAC
26	Stard7-R	GAACCTCAGAACCACTAACAGC
27	Cth-F	TTCCTGCCTAGTTTCCAGCAT
28	Cth-R	GGAAGTCCTGCTTAAATGTGGTG
29	Tmbim6-F	TCCCACATAACTCCCTCGACA
30	Tmbim6-R	GTGTGTGACCACATGGACATAG
31	Aldh5a1-F	CGGTCAAGGAGAGGAGCTTAC
32	Aldh5a1-R	GGACTAGCCCTCGCTTATCTTT
33	Neurl4-F	TACGCACATGGCCTCGTTTT
34	Neurl4-R	CCCATCACGCCTCACTTCA
35	Nrbf2-F	AAGGACCCCTCAACCTTGCT
36	Nrbf2-R	AAGGACCCCTCAACCTTGCT
37	Hspd1-F	CACAGTCCTTCGCCAGATGAG
38	Hspd1-R	CTACACCTTGAAGCATTAAGGCT
39	ANP-F	GCTTCCAGGCCATATTGGAG
40	ANP-R	GGGGGCATGACCTCATCTT
41	BNP-F	GAGGTCACTCCTATCCTCTGG
42	BNP-R	GCCATTTCCTCCGACTTTTCTC
43	Ppargc1a-F	TATGGAGTGACATAGAGTGTGCT
44	Ppargc1a-R	CCACTTCAATCCACCCAGAAAG
45	GAPDH-F	AGGTCGGTGTGAACGGATTTG
46	GAPDH-R	TGTAGACCATGTAGTTGAGGTCA
47	SKA-F	CCCAAAGCTAACCGGGAGAAG
48	SKA-R	CCAGAATCCAACACGATGCC

The List sequence of miRNA

1	miR27b-mimic	UUCACAGUGGCUAAGUUCUGC
2	miR27b-inhibitor	CGUCUUGAAUCGGUGACACUU

Regents or kits included Sirius red (Solarbio, Catalog: G1470, Beijing, China), miR-27b-3p mimic (Ribobio, Catalog: 61743, Guangzhou, China), mimic-NC (Ribobio Catalog: 61744,), miR-27b-3p inhibitor (Ribobio, Catalog: 80613), inhibitor-NC(Ribobio, Catalog: 80615), Lipofectamine 3000 regent (Thermo Fisher Scientific, Catalog: L3000015, Boston, Massachusetts, USA), DAB (Thermo Fisher Scientific, Catalog: 34002), Opti-MEM (Thermo Fisher Scientific, Catalog:31985062,), si-FGFR1(Santa Cruz Biotechnology, Catalog: sc-29317, Texas ,USA), control si-RNA(Santa Cruz Biotechnology, Catalog: sc-37007) , isoflurane (RWD Life Science, Catalog: R510-22, Shenzhen, China), AngII (Sigma-Aldrich, Catalog: 05-23-0101, Darmstadt, Germany), ISO (Sigma-Aldrich, Catalog: 1351005), DMEM (Thermo Fisher Scientific, Catalog: A4192101), Pentobarbital (Sigma-Aldrich, Catalog: P3761, Darmstadt, Germany), FBS (Gibco Life Technologies, Catalog: 10082147, Gaithersburg, MD, USA), WGA (CytoFlamma, Catalog: RCS113, Korea), Neonatal Heart Dissociation Kit (MACS, Catalog:130-098-373, Germany), Cardiac Myocyte Medium (Cell Biologics, Catalog: M1263, Chicago, USA), Mito Stress Test Kit (Agilent Technologies, Catalog:103015-100, California, USA), FGF1 ELISA kits (Bio-Techne, Catalog: DY4686-05, Minnesota, USA), BrdU (Selleck Chemicals, Catalog: S791895, Texas, USA). Antibodies included BNP (abcam, Catalog: ab19645, Cambridge Science Park, UK), ANP32E (abcam, Catalog: ab5993), FGF1 (abcam, Catalog: ab207321), Smad3 (Cell Signaling Technology CST, Catalog: #9523), Phospho-Smad3 (CST, Catalog: #9520), GAPDH (Zhong Shan Golden Bridge ZSGB, Catalog: TA309157, Beijing, China), PGC1α (abcam, Catalog: ab54481), PGC1β (abcam, Catalog: ab176328), FGFR1 (abcam, Catalog: ab206328), Mac-2(Santa Cruz Biotechnology, Catalog: sc-32790, Texas ,USA), IL-1β (abcam, Catalog: ab9722), NF-κB p65 (abcam, Catalog:ab19870).

Table S2 Baseline characteristics of control subjects and enrolled HCM patients

	Donor Control (n=6)	HCM Patients (n=6)	P-value
**Clinical/demographic features**			
Male, n（%）	6 (100)	5 (83)	1
Age, years	52±6.83	53±3.88	0.7367
Heart function classification, n（%）			
NYHA class I	6 (100)	0	<0.0001
NYHA class II	0	0	
NYHA class III	0	6 (100)	<0.0001
Family history of HCM	NO	NO	
Hypertension, n（%）	0 (0)	6 (100)	<0.0001
**Drug treatment**			
β-blockers	NO	YES	<0.0001
Calcium channel blockers	NO	YES	<0.0001

NYHA：New York Heart Association

SUPPLMENTAL DATA: Differential expression genes between Ctrl/TAC and KO/TAC

Gene ID	Gene_symbol	P.Value	FC	Regulation
11304	Abca4	0.020769594	2.765765766	up
11306	Abcb7	0.042373276	1.440286624	up
11307	Abcg1	0.043718034	-1.20649652	down
11363	Acadl	0.037824265	1.377587033	up
11364	Acadm	0.027082118	1.633563166	up
11370	Acadvl	0.033350839	1.490936697	up
11421	Ace	0.006838616	-2.313914881	down
11492	Adam19	0.018017676	-2.434451902	down
11504	Adamts1	0.042441427	-1.652443755	down
11607	Agtr1a	0.049653102	1.71871714	up
11622	Ahr	0.004912152	1.940350877	up
11641	Akap2	0.031445582	-1.554529717	down
11651	Akt1	0.008964881	-1.348158131	down
11668	Aldh1a1	0.005510345	-2.101957071	down
11677	Akr1b3	0.021660505	1.348249684	up
11684	Alox12	0.009124551	1.96039604	up
11722	Amy1	0.044529311	2.016620499	up
11757	Prdx3	0.012815867	1.264809301	up
11774	Ap3b1	0.006312238	-1.211545231	down
11785	Apbb1	0.029966218	1.416741162	up
11787	Apbb2	0.040377812	1.261970716	up
11807	Apoa2	0.021615574	1.747989276	up
11820	App	0.030631421	-1.284752654	down
11832	Aqp7	0.047912394	1.886749198	up
11854	Rhod	0.046754019	-1.975806452	down
11867	Arpc1b	0.034454598	-1.201342282	down
11881	Arsb	0.0100518	-1.563411079	down
11911	Atf4	0.015385441	-1.550769231	down
11927	Atox1	0.01670868	-1.364669902	down
11946	Atp5a1	0.037466274	1.238179056	up
11947	Atp5b	0.010363123	1.205945037	up
11949	Atp5c1	0.003115826	1.264777639	up
11950	Atp5f1	0.009258548	1.457141984	up
11981	Atp9a	0.010797221	-1.214978385	down
12033	Bcap29	0.020796256	1.643354083	up
12040	Bckdhb	0.003351791	1.617180095	up
12050	Bcl2l2	0.012268825	-1.285787321	down
12051	Bcl3	0.040893078	-2.130387931	down
12054	Bcl7b	0.014884457	-1.229494441	down
12111	Bgn	0.000675212	-2.355314274	down
12125	Bcl2l11	0.022748193	1.576822917	up
12153	Bmp1	0.024388706	-1.419223459	down
12159	Bmp4	0.012062776	-1.4	down
12257	Tspo	0.005531836	-1.649084249	down
12258	Serping1	0.015650562	-1.821284706	down
12262	C1qc	0.033706282	-1.214301239	down
12268	C4b	0.012060854	-2.5395189	down

12273	C5ar1	0.025483694	-1.648678414	down
12292	Cacna1s	0.028747671	1.807302231	up
12306	Anxa2	0.016924595	-1.389582923	down
12335	Capn3	0.028618114	2.387096774	up
12348	Car11	0.039308759	-2.365243004	down
12388	Ctnnd1	0.036372418	-1.226836232	down
12406	Serpinh1	0.039079971	-1.621337318	down
12477	Ctla4	0.027608589	3.358108108	up
12487	Cd28	0.0077047	2.185840708	up
12496	Entpd2	0.025286583	-1.494389027	down
12497	Entpd6	0.024687178	1.370592949	up
12499	Entpd5	0.014865552	1.641700231	up
12509	Cd59a	0.029446932	1.389422417	up
12512	Cd63	0.009937767	-1.652539615	down
12522	Cd83	0.015879978	-1.340320592	down
12544	Cdc45	0.015869762	1.363636364	up
12589	Ift81	0.016062341	1.755208333	up
12609	Cebpd	0.011184722	-2.411733333	down
12651	Chkb	0.043448793	1.331686989	up
12702	Socs3	0.036100737	-1.899470899	down
12774	Ccr5	0.037846945	2.496323529	up
12790	Cnga3	0.012323708	2.547619048	up
12795	Plk3	0.023260885	-1.495633188	down
12808	Cobl	0.004228198	-1.408921933	down
12829	Col4a4	0.041745505	-1.759493671	down
12830	Col4a5	0.004175533	-1.541645376	down
12832	Col5a2	0.021942195	-2.169245648	down
12833	Col6a1	0.035355987	-1.460520095	down
12834	Col6a2	0.047380854	-1.452855382	down
12842	Col1a1	0.017794104	-1.771174206	down
12843	Col1a2	0.00187376	-2.178441747	down
12845	Comp	5.68754E-05	-8.505681818	down
12850	Coq7	0.010985786	1.305556801	up
12858	Cox5a	0.004722369	1.351403582	up
12859	Cox5b	0.006275471	1.263026477	up
12865	Cox7a1	0.00892567	1.436673862	up
12890	Cplx2	0.030432495	-1.295983087	down
12905	Cradd	0.014575408	1.342982456	up
12915	Atf6b	0.032592582	-1.315086782	down
12931	Crlf1	0.007865537	-9.140794224	down
12952	Cry1	0.024235568	-1.847068146	down
12959	Cryba4	0.004353499	2.69858871	up
12971	Crym	0.039542853	-5.404761905	down
12974	Cs	0.036285397	1.242009646	up
12978	Csf1r	0.043650172	-1.244377811	down
12984	Csf2rb2	0.048189233	-2.077922078	down
13043	Cttn	0.002625828	-1.440605072	down
13056	Cyb561	0.006076001	-1.318885449	down
13063	Cycs	0.035887192	1.293329811	up

13076	Cyp1a1	0.026403246	3.798449612	up
13120	Cyp4b1	0.049345018	1.521727395	up
13139	Dgka	0.007458008	1.305365297	up
13143	Dapk2	0.002001372	-1.742630385	down
13171	Dbt	0.01073441	1.708362129	up
13177	Eci1	0.016082722	1.708419096	up
13180	Pcbd1	0.036701169	-2.308724832	down
13197	Gadd45a	0.032851795	1.806075739	up
13202	Ddt	0.000885463	1.376059247	up
13382	Dld	0.042347488	1.528099437	up
13385	Dlg4	0.049624466	-1.739932886	down
13401	Dmwd	0.038440954	-1.225534673	down
13419	Dnase1	0.01420294	1.627737226	up
13429	Dnm1	0.003705223	-1.900501672	down
13448	Dok1	0.008527638	-1.730563003	down
13481	Dpm2	0.00921492	-1.222907036	down
13498	Atn1	0.034053292	-1.416821994	down
13557	E2f3	0.041634284	-1.359163592	down
13614	Edn1	0.041950854	-1.592169657	down
13636	Efna1	0.024403178	1.289966489	up
13650	Rhbdf1	0.000999077	-1.366962546	down
13654	Egr2	0.033431122	-8.763975155	down
13655	Egr3	0.024698822	-4.855263158	down
13666	Eif2ak3	0.021788939	-1.278709677	down
13717	Eln	0.003142336	-2.757206208	down
13804	Endog	0.024205886	1.297805948	up
13808	Eno3	0.021246807	1.420115743	up
13822	Epb41l2	0.025143572	-1.285870158	down
13848	Ephb6	0.033244892	-2.771929825	down
13850	Ephx2	0.028625998	1.67013373	up
13992	Khdrbs3	2.59629E-05	1.414452075	up
14011	Etv6	0.011475177	-1.271672772	down
14081	Acsl1	0.045284771	1.437119496	up
14085	Fah	0.009641114	2.256421419	up
14102	Fas	0.046654224	1.612487611	up
14107	Fat1	0.019634578	-1.922005571	down
14115	Fbln2	0.010244218	-1.974538032	down
14120	Fbp2	0.018289024	2.625392396	up
14156	Fen1	0.026776286	1.394021739	up
14164	Fgf1	0.00576768	1.430165685	up
14182	Fgfr1	0.037323731	-1.95574086	down
14194	Fh1	0.011354744	1.328539489	up
14199	Fhl1	0.026317952	-1.868884307	down
14200	Fhl2	0.02542978	2.132138864	up
14219	Ctgf	0.009401549	-9.45902537	down
14232	Fkbp8	0.002237396	-1.224795933	down
14234	Foxc2	0.049531575	-3.334710744	down
14239	Foxs1	0.012893405	-2.253731343	down
14264	Fmod	0.015860953	-7.77262181	down

14268	Fn1	0.014936086	-2.285427807	down
14314	Fstl1	0.042550777	-1.99983632	down
14345	Fut4	0.041791637	2.409090909	up
14362	Fzd1	0.001372592	-1.865965834	down
14373	G0s2	0.012545844	-1.394438555	down
14385	Slc37a4	0.039642143	1.214309821	up
14457	Gas7	0.027186209	-1.481693364	down
14467	Gbas	0.036608568	1.290615512	up
14605	Tsc22d3	0.02363199	-1.346649871	down
14633	Gli2	0.03974974	-1.84	down
14661	Glud1	0.023953036	1.209646576	up
14674	Gna13	0.041247991	-1.295	down
14711	Gnmt	0.017461905	2.326589595	up
14726	Pdpn	0.027513093	-2.428726877	down
14747	Cmklr1	0.038116643	-2.061915511	down
14775	Gpx1	0.007688225	-1.63417707	down
14789	P3h3	0.019913396	-1.809976247	down
14791	Emg1	0.017524139	1.237646002	up
14816	Grm1	0.034957537	1.428466077	up
14874	Gstz1	0.024875754	1.386986751	up
14998	H2-DMa	0.020995614	1.73046875	up
15107	Hadh	0.028243469	1.635933252	up
15108	Hsd17b10	0.04691161	1.31227281	up
15159	Hccs	0.031540689	1.292881484	up
15191	Hdgf	0.005874749	-1.219111635	down
15205	Hes1	0.018082627	1.445655211	up
15211	Hexa	0.011452374	-1.352361924	down
15214	Hey2	0.036885552	1.58013544	up
15275	Hk1	0.007544948	-1.334356016	down
15331	Hmgn2	0.021050057	1.320014258	up
15374	Hn1	0.022066998	-1.573488206	down
15410	Hoxb3	0.026172043	-1.887096774	down
15464	Hrc	0.03427562	1.545531736	up
15468	Prmt2	0.012817498	-1.457586207	down
15490	Hsd17b7	0.038012693	1.498936924	up
15510	Hspd1	0.032243552	1.482700117	up
15926	Idh1	0.000271435	1.464520986	up
15937	Ier3	0.036295928	-1.860644519	down
16145	Igtp	0.016685478	2.839416058	up
16168	Il15	0.022752092	2.097920696	up
16195	Il6st	0.025846622	-1.399644497	down
16373	Irx3	0.016506777	2.442497262	up
16402	Itga5	0.000431047	-1.733495418	down
16419	Itgb5	0.002014463	-1.382397343	down
16420	Itgb6	0.000817063	1.961251863	up
16425	Itih2	0.04381889	-3.755555556	down
16428	Itk	0.014680125	1.858426966	up
16468	Jarid2	0.018718819	1.408783784	up
16477	Junb	0.027241781	-2.28765462	down

16518	Kcnj2	0.009512771	2.03768624	up
16560	Kif1a	0.012417352	-3.692307692	down
16570	Kif3c	0.013271313	-1.266447368	down
16581	Kifc2	0.044914908	1.206730769	up
16593	Klc1	0.003474354	-1.361409323	down
16600	Klf4	0.036523095	-1.432121212	down
16618	Klk1b26	0.003425312	8.157894737	up
16668	Krt18	0.005951144	-9.311111111	down
16716	Ky	0.002184179	3.728542914	up
16803	Lbp	6.43603E-05	-2.049856184	down
16832	Ldhb	0.018871933	1.506327445	up
16855	Lgals4	0.011282421	2.752462527	up
16885	Limk1	0.04527493	-1.278106509	down
16909	Lmo2	0.027902765	-1.600847757	down
16922	Phyh	0.006885363	1.204183276	up
16949	Loxl1	0.007121753	-2.020205945	down
16971	Lrp1	0.002850607	-2.082506654	down
16998	Ltbp3	0.001356439	-1.898951138	down
17025	Alad	0.021644436	1.419041534	up
17117	Amacr	0.006999066	1.408386638	up
17130	Smad6	0.016324975	-1.889196676	down
17131	Smad7	0.033027003	-1.391534392	down
17133	Maff	7.35793E-05	-2.111944605	down
17134	Mafg	0.042119052	-1.270107742	down
17135	Mafk	0.042097124	-1.892606868	down
17150	Mfap2	0.016828692	-1.935704514	down
17160	Man2b2	0.039854843	-1.270177296	down
17192	Mbd3	0.019938537	-1.29017882	down
17220	Mcm7	0.02862861	1.206216831	up
17254	Slc3a2	0.004422881	-1.374470018	down
17256	Mea1	0.010077434	-1.203089615	down
17285	Meox1	0.036231091	-3.14378921	down
17299	Mettl1	0.023360239	-1.622674934	down
17304	Mfge8	0.011559774	-1.226454657	down
17313	Mgp	0.032016059	-2.55532467	down
17314	Mgmt	0.00790399	1.655084313	up
17342	Mitf	0.004390843	1.448539971	up
17380	Mme	0.049789082	2.038216561	up
17390	Mmp2	0.001901863	-2.240243161	down
17532	Mras	0.008625346	-1.413369714	down
17534	Mrc2	0.025352115	-2.04121965	down
17684	Cited2	0.039273133	1.563963112	up
17755	Map1b	0.037896676	-1.76146789	down
17769	Mthfr	0.016123693	-1.399795501	down
17846	Commd1	0.003730136	1.282025881	up
17872	Ppp1r15a	0.000108785	-1.949029126	down
17873	Gadd45b	5.30113E-05	1.626079447	up
17904	Myl6	0.04864208	-1.297495418	down
17965	Nbl1	0.008451404	-2.112410987	down

17972	Ncf4	0.045088564	1.367713004	up
17992	Ndufa4	0.032194193	1.307827862	up
17993	Ndufs4	0.009816641	1.523334828	up
18016	Nf2	0.04013468	-1.244906932	down
18019	Nfatc2	0.014174601	1.41729639	up
18024	Nfe2l2	0.043313571	-1.227411858	down
18033	Nfkb1	0.018110647	-1.252310062	down
18034	Nfkb2	0.017560479	-1.33697398	down
18044	Nfya	0.0123523	-1.347314578	down
18081	Ninj1	0.003958376	-1.235768345	down
18091	Nkx2-5	0.035580363	-1.229206963	down
18100	Mrpl40	0.004176435	1.273265196	up
18105	Nqo2	0.009047041	1.488235294	up
18113	Nnmt	0.00077924	-1.510638298	down
18115	Nnt	0.001073647	2.734572017	up
18158	Nppb	0.036536583	-4.085167341	down
18207	Nthl1	0.033301642	1.532	up
18220	Nucb1	0.015154444	-1.275865941	down
18226	Nup62	0.001634285	-1.349740933	down
18451	P4ha1	0.029328311	-2.147669706	down
18542	Pcolce	0.000762733	-1.805675963	down
18551	Pcsk4	0.033962635	2.572815534	up
18552	Pcsk5	0.004618966	-1.939534884	down
18582	Pde6d	0.043490519	-1.222609983	down
18585	Pde9a	0.043870342	-1.616224649	down
18595	Pdgfra	0.007621187	-1.543509966	down
18607	Pdpk1	0.003297324	-1.220521307	down
18611	Pea15a	0.009257323	-1.28524366	down
18639	Pfkfb1	0.044479335	5.508305648	up
18647	Cdk14	0.0121421	-1.732265446	down
18648	Pgam1	0.002349505	-1.426286037	down
18674	Slc25a3	0.005014083	1.291419412	up
18739	Pitpnm1	0.014815712	-1.33843717	down
18759	Prkci	0.000385574	-1.357965451	down
18761	Prkcq	0.025842435	2.233802817	up
18767	Pkia	0.035481996	1.502082448	up
18793	Plaur	0.025650438	-1.72361809	down
18807	Pld3	0.005248744	-1.396136314	down
18821	Pln	0.008816456	1.791403927	up
18858	Pmp22	0.018129714	-1.597399004	down
18950	Pnp	0.023419175	-1.342368641	down
18951	5-Sep	0.000735201	-2.061010486	down
19017	Ppargc1a	0.032221113	1.242105263	up
19158	Cyth2	0.000581678	-1.226259131	down
19186	Psme1	0.0068049	1.546316891	up
19188	Psme2	0.047372527	1.22268098	up
19207	Ptch2	0.04347312	-1.73006135	down
19223	Ptgis	0.049182278	-2.238151659	down
19246	Ptpn1	0.007446972	-1.41855304	down

19268	Ptprf	0.03839975	-2.084639498	down
19288	Ptx3	0.014033211	-2.741573034	down
19299	Abcd3	0.032704735	1.535362935	up
19301	Pxmp2	0.00058582	1.48850865	up
19328	Rab12	0.024214408	1.209736537	up
19329	Rab17	0.020543574	1.912878788	up
19335	Rab23	0.046082612	-1.449554896	down
19378	Aldh1a2	0.029314133	-3.628719276	down
19655	Rbmx	0.007723088	1.229652145	up
19659	Rbp1	0.045907083	-3.130499259	down
19697	Rela	0.009238586	-1.201421443	down
19882	Mst1r	0.044869161	-2.066225166	down
20130	Rras	0.014344994	-1.352279229	down
20163	Rsu1	0.0464283	-1.248649438	down
20166	Rtkn	0.012121613	-1.714285714	down
20183	Rxrg	0.043493504	1.454621427	up
20195	S100a11	0.040996806	-1.402728227	down
20196	S100a13	0.030645335	-1.274575621	down
20204	Prrx2	0.010066566	-4.439393939	down
20292	Ccl11	0.013127495	3.280373832	up
20293	Ccl12	0.030541968	-2.371819961	down
20296	Ccl2	0.023067361	-2.136631331	down
20317	Serpinf1	0.010099686	-2.377748168	down
20319	Sfrp2	0.002548962	-4.100952381	down
20364	Sepw1	0.001906172	-1.297732577	down
20377	Sfrp1	0.02461348	-1.857142857	down
20382	Srsf2	0.004273003	1.224974312	up
20384	Srsf5	0.038362781	1.7018414	up
20397	Sgpl1	0.016024013	-1.271053662	down
20405	Sh3gl1	0.012277609	-1.377445749	down
20410	Sorbs3	0.004212921	-1.354597292	down
20442	St3gal1	0.026217039	-1.209401709	down
20444	St3gal2	0.041217667	-1.266666667	down
20448	St6galnac4	0.044993054	-1.221762048	down
20452	St8sia4	0.014511801	2.117408907	up
20481	Ski	0.020935325	-1.498602325	down
20482	Skil	0.001132282	-1.485232068	down
20512	Slc1a3	0.031417457	-2.329479769	down
20598	Smpd2	0.002801617	1.415715622	up
20613	Snai1	0.021242297	-2.07980653	down
20656	Sod2	0.025034632	1.380103787	up
20657	Sod3	0.005303038	-1.466046191	down
20682	Sox9	0.007169909	-3.443877551	down
20698	Sphk1	0.001455228	-2.761538462	down
20715	Serpina3g	0.042510801	2.510948905	up
20716	Serpina3n	0.035528451	-3.208090379	down
20720	Serpine2	0.008625587	-1.702135493	down
20729	Spin1	0.025454657	-1.249765552	down
20807	Srf	0.004531921	-1.498136955	down

20810	Srm	0.006817185	-1.282329432	down
20848	Stat3	0.001748943	-1.267526006	down
20852	Stat6	0.03843167	-1.228724194	down
20907	Stx1a	0.021108417	-1.899390244	down
20916	Sucla2	0.038467362	1.662046859	up
20969	Sdc1	0.036659079	-1.535051546	down
20972	Syngr1	0.010288081	-1.364031487	down
20975	Synj2	0.044552426	1.471495456	up
21346	Tagln2	0.004906357	-1.429698448	down
21412	Tcf21	0.040112735	-1.890625	down
21648	Dynlt1b	0.00435392	2.999627005	up
21664	Phlda1	0.010486751	-2.046327684	down
21754	Tesk1	0.018819391	-1.239674868	down
21766	Tex261	0.015400692	-1.254862667	down
21767	Tex264	0.024160574	-1.223048812	down
21803	Tgfb1	0.005450134	-1.328423637	down
21810	Tgfbi	0.026387199	-1.386931155	down
21813	Tgfbr2	0.032296421	-1.45308095	down
21815	Tgif1	0.011232236	-1.621407334	down
21817	Tgm2	0.000380099	-1.64386936	down
21821	Ift88	0.042952125	1.429435484	up
21825	Thbs1	0.046152043	-4.44403444	down
21827	Thbs3	0.003842221	-2.227578475	down
21848	Trim24	0.026623226	1.23977824	up
21887	Tle3	0.01799229	-1.858695652	down
21894	Tln1	0.015270257	-1.290272774	down
21937	Tnfrsf1a	0.017218981	-1.29310952	down
21938	Tnfrsf1b	0.041771429	-1.516333938	down
21987	Tpd52l1	0.048402823	1.773345422	up
22031	Traf3	0.004601236	-1.367123288	down
22121	Rpl13a	0.032292713	-1.235997899	down
22142	Tuba1a	0.005631928	-1.493429487	down
22145	Tuba4a	0.003219964	1.581595195	up
22153	Tubb4a	0.041232752	-1.325626204	down
22154	Tubb5	0.048778235	-1.260525394	down
22160	Twist1	0.0352784	-2.070224719	down
22210	Ube2b	0.048397415	1.327081331	up
22213	Ube2g2	0.013519674	-1.200929503	down
22232	Slc35a2	0.019903736	-1.224662162	down
22240	Dpysl3	0.015988134	-1.844880027	down
22275	Urod	0.008590417	1.202281306	up
22276	Uros	0.01230108	1.202609641	up
22323	Vasp	0.019730269	-1.2948903	down
22342	Lin7b	0.005725886	-2.951612903	down
22352	Vim	0.025118893	-1.597122302	down
22401	Zmat3	0.043917403	-1.205748865	down
22403	Wisp2	0.047188711	-3.575633688	down
22431	Wt1	0.001693267	-1.866666667	down
22436	Xdh	0.029914347	1.587245233	up

22601	Yap1	0.000320657	-1.308951407	down
22629	Ywhah	0.019756736	-1.286872237	down
22673	Zfp185	0.012748829	-4.119402985	down
22680	Zfp207	0.018385367	-1.229934641	down
22682	Zfand5	0.032949572	1.210685649	up
22690	Zfp28	0.005493151	1.322775264	up
22704	Zfp46	0.014945413	1.31260283	up
22759	Zfp97	0.03125254	1.841549296	up
22761	Zfpm1	0.000845039	-1.44808232	down
22793	Zyx	0.002867382	-1.732332516	down
23805	Apc2	0.015120616	-1.796052632	down
23831	Car14	0.020332345	1.582842883	up
23876	Fbln5	0.035439403	-1.448979592	down
23879	Fxr2	0.002742345	-1.260559151	down
23887	Ggt5	0.004387968	1.430120482	up
23950	Dnajb6	0.027277098	-1.303612059	down
23965	Tenm3	0.030863508	-1.805194805	down
23971	Papss1	0.046499519	-1.268933539	down
23986	Eci2	0.007830887	1.38525915	up
23991	Cib1	0.00197051	-1.283068783	down
26398	Map2k4	0.042433493	1.202579497	up
26411	Map4k1	0.048224372	1.8125	up
26412	Map4k2	0.043022999	1.24124953	up
26433	Plod3	0.003702684	-1.294287482	down
26549	Itgb1bp2	0.021846609	1.218013052	up
26558	Homer3	0.001641257	-1.557655955	down
26561	Mmp23	0.000371785	-1.923491379	down
26900	Ddx3y	0.024071344	1.460266667	up
26926	Aifm1	0.005749946	1.203847959	up
26949	Vat1	0.004887074	-1.366324363	down
26968	Islr	0.006566143	-1.814918492	down
27008	Micall1	0.016896189	-1.315211907	down
27059	Sh3d19	0.002895016	-1.585814361	down
27280	Phlda3	0.000609486	-2.134973005	down
27368	Tbl2	0.014028787	-1.234917733	down
27369	Dguok	0.006887139	1.319375574	up
27393	Mrpl39	0.000693481	1.300142023	up
27425	Atp5l	0.009310866	1.261839984	up
27632	Nelfe	0.034809314	-1.210175812	down
27883	Tango2	0.025021236	1.2350776	up
28080	Atp5o	0.001181488	1.259469666	up
28081	Fam104a	0.001106693	-1.222408427	down
29817	Igfbp7	0.037661813	-1.732055366	down
30878	Apln	0.010381812	1.829232996	up
30928	Zbtb18	0.008622236	1.228087986	up
30939	Pttg1	0.001062878	1.284304582	up
50498	Ebi3	0.014023912	2.022058824	up
50709	Hist1h1e	0.008321466	1.881355932	up
50874	Tmod4	0.038881444	1.485313932	up

50915	Grb14	0.001603227	1.560271882	up
51793	Ddah2	0.006244179	-1.525444023	down
51795	Srpx	0.037708412	-2.740495868	down
51798	Ech1	0.018762371	1.883687293	up
51812	Mcrs1	0.025842837	1.261838893	up
51885	Tubgcp4	0.014882986	-1.222306525	down
51902	Rnf24	0.013670712	-1.421052632	down
52250	Reep1	0.047574647	1.228479853	up
52357	Wwc2	0.013528782	-1.320495646	down
52377	Rcn3	0.03883724	-1.871004712	down
52430	Echdc2	0.027075038	1.257905544	up
52502	Carhsp1	0.004351219	-1.935558113	down
52538	Acaa2	0.013601909	1.894330761	up
52633	Nit2	0.014193937	1.407011543	up
52685	Cd300lg	0.020002323	1.765492322	up
52840	Dbndd2	0.008013529	-1.32145305	down
52856	Mtg2	0.013603507	1.35742732	up
52898	Rnasek	0.029184975	-1.205634522	down
53412	Ppp1r3c	0.01953106	-1.702284022	down
53422	Ybx2	0.037171	1.681422925	up
53859	Map3k14	0.002696444	-1.756388416	down
53871	Pkd2l2	0.02228667	1.402390438	up
53895	Clpp	0.02422334	1.270616048	up
54325	Elovl1	0.003543216	-1.233127185	down
54353	Skap2	0.04176706	-1.298139535	down
54383	Phc2	0.017952931	-1.300991736	down
54396	Irgm2	0.035818615	2.162589037	up
54667	Atp8b2	0.00671253	-1.547747748	down
55950	Bri3	0.00367423	-1.318155692	down
55983	Pdzrn3	0.029785086	-1.701705855	down
55984	Camkk1	0.033932341	-2.348547718	down
55988	Snx12	0.008746667	-1.259496284	down
56043	Akr1e1	0.03606983	1.37715959	up
56077	Dgke	0.028940732	1.39578714	up
56088	Psmg1	0.000439531	1.328606249	up
56198	Heyl	0.004227484	-1.759272218	down
56218	Patz1	0.013283874	1.347328244	up
56222	Cited4	0.030313131	-1.496626768	down
56277	Tmem45a	0.009109842	-1.659883721	down
56284	Mrpl19	0.012205937	1.31557585	up
56294	Ptpn9	0.005717461	-1.400873839	down
56309	Mycbp	0.036108128	1.242956458	up
56312	Nupr1	0.014070881	-2.223542945	down
56320	Dbn1	0.005254339	-1.975360577	down
56336	B4galt5	0.000350623	-1.880766501	down
56369	Apip	0.006308981	1.374119718	up
56418	Ykt6	0.010630733	-1.209578452	down
56428	Mtch2	0.03386294	1.203946655	up
56451	Suclg1	0.00592986	1.412861553	up

56455	Dynll1	0.014348901	-1.621379454	down
56541	Habp4	0.003161575	-1.201147567	down
56620	Clec4n	0.048049576	-1.648648649	down
56626	Poll	0.030182756	1.228654971	up
56631	Trim17	0.045734452	-1.655940594	down
56711	Plag1	0.035041858	1.710344828	up
56715	Rabgef1	0.004715869	-1.288637194	down
56722	Litaf	0.003575887	-1.534218289	down
56742	Psrc1	0.019694368	-2.764705882	down
56752	Aldh9a1	0.014692499	1.702942017	up
56847	Aldh1a3	0.023075687	-2.841463415	down
57080	Gtf2ird1	0.032612089	-1.329793879	down
57246	Tbx20	0.040164418	-1.240362285	down
57260	Ltb4r2	0.00037968	1.602941176	up
57312	Mrps31	0.008223637	1.34389562	up
57344	As3mt	0.001355068	1.454730832	up
57436	Gabarapl1	0.025407456	-1.444903581	down
57743	Sec61a2	0.020496126	1.244863014	up
57764	Ntn4	0.045943561	-1.468275246	down
57783	Tnip1	0.010884962	-1.620767836	down
57912	Cdc42se1	0.024376489	-1.267014406	down
58223	Mmp19	0.037446649	-1.576177285	down
58233	Dnaja4	0.003202647	-1.386787538	down
58235	Pvrl1	0.046911806	-1.897435897	down
58244	Stx6	0.001249672	-1.498202732	down
58799	Crbn	0.049752898	1.364464692	up
58875	Hibadh	0.00512233	1.221429848	up
59036	Dact1	0.021621764	-1.405405405	down
59083	Fetub	0.032144749	-2.254166667	down
59095	Fxyd6	0.028640368	-1.437523594	down
59126	Nek6	0.007843662	-2.202409639	down
60440	Iigp1	0.005360744	2.58168028	up
60595	Actn4	0.014540818	-1.390252017	down
60613	Kcnq4	0.022383348	1.787810384	up
63953	Dusp10	0.005481704	1.508124077	up
63955	Cables1	0.02903196	-1.434610304	down
64138	Ctsz	0.008616462	-1.40704698	down
64144	Mllt1	0.048707178	-1.245598592	down
64291	Osbpl1a	0.005442204	1.205444549	up
64659	Mrps14	0.048589251	1.337706904	up
65112	Pmepa1	0.01456599	-1.733149171	down
65115	Bean1	0.049243573	-1.845771144	down
65964	Zak	0.002505204	1.283504449	up
66046	Ndufb5	0.013428988	1.342826763	up
66086	Fopnl	0.037379276	1.200580341	up
66091	Ndufa3	0.01624082	1.283138815	up
66108	Ndufa9	0.037228349	1.302739737	up
66125	Sf3b5	0.01406094	-1.202344855	down
66132	1110008L16Rik	0.004546811	1.431818182	up

66148	Dnajc15	0.020768805	1.432791184	up
66151	Prr13	0.001315117	-1.257180402	down
66168	Grina	0.008895808	-1.225849169	down
66174	Nudt14	0.018646221	1.566119901	up
66211	Rpl3l	0.045161811	2.071055901	up
66218	Ndufb9	0.009304076	1.205241408	up
66230	Mrps28	0.001418651	1.253660131	up
66246	Osgep	0.01232994	1.355984431	up
66251	Arfgap3	0.022675934	-1.633069083	down
66278	Smim20	0.006022013	1.397986999	up
66313	Smurf2	0.005702305	-1.325253256	down
66349	Atp5sl	0.023432712	1.241526401	up
66399	Tsfm	0.014362222	1.215323646	up
66400	Alkbh7	0.029218057	1.370382751	up
66407	Mrps15	0.036616046	1.238918476	up
66427	Cyb5b	0.015537315	1.208137432	up
66494	Prelid1	0.027503226	-1.207474677	down
66497	Cmss1	0.026022558	1.602799572	up
66498	Dda1	0.001155619	-1.322135417	down
66513	Tab1	0.00234695	1.501766784	up
66515	Cul7	0.022155005	-1.249524715	down
66556	Drap1	0.010285907	-1.258068563	down
66559	Metap1d	0.017577354	1.275405557	up
66616	Snx9	0.03563342	-1.205015045	down
66671	Ccnh	0.036041997	1.374624374	up
66694	Uqcrfs1	0.006270104	1.333236683	up
66701	Spryd4	0.04289129	1.331673484	up
66705	Dnase1l2	0.015846203	-1.674757282	down
66706	Ndufaf3	0.031505614	1.250916627	up
66832	Rsph3a	0.000115681	-1.689481066	down
66841	Etfdh	0.01143122	1.588355884	up
66855	Tcf25	0.00829942	-1.219635982	down
66898	Baiap2l1	0.00155139	-1.654558933	down
66910	Tmem107	0.003950872	-1.424920128	down
66945	Sdha	0.015166661	1.363172885	up
66993	Smarcd3	0.005485891	-1.248420221	down
67003	Uqcrc2	0.040188769	1.348554245	up
67013	Oma1	0.005080718	1.424832496	up
67031	Upf3a	0.006532391	-1.396313364	down
67036	Mrpl45	0.002218078	1.478130733	up
67134	Nop56	0.02459921	1.281818182	up
67164	Lipt2	0.010944482	1.310454908	up
67168	Lpar6	0.015653831	1.539325843	up
67171	Dram2	0.049663156	1.594275254	up
67196	Ube2t	0.016281572	1.727129338	up
67220	Plekho1	0.048966999	-1.657608369	down
67231	Tbc1d20	0.001738991	-1.219258177	down
67264	Ndufb8	0.023724771	1.266254769	up
67269	Agtpbp1	0.01709867	1.376762067	up

67308	Mrpl46	0.032011127	1.43658827	up
67383	Carnmt1	0.009897765	1.372352941	up
67414	Mfn1	0.011801607	1.210721724	up
67425	Eps8l1	0.043752356	-1.664259928	down
67426	Adck3	0.03440671	1.326518769	up
67440	Mtpap	0.022729503	1.298959747	up
67460	Decr1	0.005031022	1.64975485	up
67484	Eepd1	0.024220685	1.659030837	up
67492	Zfand4	0.031190211	1.68	up
67525	Trim62	0.030788263	-1.969026549	down
67528	Nudt7	0.013175994	1.709123526	up
67542	Cog6	0.014739137	1.279380068	up
67603	Dusp6	0.031891416	-1.481371088	down
67621	Bend5	0.032365141	1.351131222	up
67655	Ctdp1	0.008466665	-1.229329173	down
67680	Sdhb	0.015717295	1.309396271	up
67681	Mrpl18	0.026528986	1.281233561	up
67704	1810037I17Rik	0.049246945	-1.320844546	down
67774	Borcs5	0.001374021	1.394888179	up
67834	Idh3a	0.003619577	1.2836653	up
67856	Echdc3	0.01328649	1.358688226	up
67861	Akr1b10	0.02113134	1.215727542	up
67893	Tmem86a	0.031344583	1.389100127	up
67993	Nudt12	0.003670781	1.382959641	up
67997	Ddx59	0.036764651	1.278877888	up
68017	Ftsj2	0.013900387	1.266248319	up
68034	Fam122a	0.022650522	-1.25259284	down
68044	Chac2	0.020900977	1.31408661	up
68115	9430016H08Rik	0.00425122	1.29255883	up
68176	Fam212a	0.040013983	1.452879581	up
68194	Ndufb4	0.029084924	1.216479395	up
68198	Ndufb2	0.000703005	1.21161888	up
68226	Efcab2	0.039518467	1.406626506	up
68267	Slc25a22	0.038843899	1.579757976	up
68316	Apoo	0.042336377	1.39834698	up
68337	Crip2	0.028852841	1.355308896	up
68342	Ndufb10	0.007310527	1.262921156	up
68349	Ndufs3	0.020626496	1.33483424	up
68440	Dusp23	0.031792262	1.537305699	up
68480	Card19	0.020882404	-1.265988957	down
68549	Sgol2a	0.009861904	2.673469388	up
68550	Tefm	0.021931606	1.342307692	up
68564	Nufip2	0.016917617	-1.3438949	down
68585	Rtn4	0.019733445	-1.351515152	down
68617	Mtcl1	0.033883977	-1.475177305	down
68646	Nadk2	0.018979231	1.495500562	up
68693	Hnrnpul2	0.015265649	-1.324632739	down
68724	Arl8a	0.004612996	-1.369590258	down
68732	Lrrc16a	0.021345636	-1.564220183	down

68770	Phtf2	0.042351276	1.333913476	up
68774	Ms4a6d	0.021722736	-1.798882682	down
68797	Pdgfrl	0.00220855	-2.585434174	down
68828	Sync	0.006842463	-1.300226586	down
68837	Foxk2	0.001291116	-1.232178415	down
68867	Rnf122	0.001393261	1.6725	up
68874	Klhdc9	0.029207811	1.244267198	up
68920	1110065P20Rik	0.042841763	-1.472714633	down
68968	Cdan1	0.037226336	1.209847597	up
69053	1810013L24Rik	0.005078553	1.257731959	up
69065	Chac1	0.015781024	2.323838081	up
69101	Ydjc	0.047976447	-2.213270142	down
69149	Kbtbd3	0.04979417	1.446348061	up
69159	Rhebl1	0.004203868	2.125	up
69163	Mrpl44	0.015720439	1.266857963	up
69202	Ptms	0.027790498	-1.873210526	down
69217	Plekha4	0.00480533	-2.166015625	down
69263	Rfc3	0.00641825	1.366127024	up
69270	Gins1	0.047837274	1.955128205	up
69408	Dnajc17	0.005675374	1.276422764	up
69487	Ndufaf5	0.026375829	1.288241074	up
69538	Antxr1	0.01260556	-1.659863946	down
69546	Mapk1ip1	0.010624017	1.22522293	up
69574	Cmbl	0.022272664	1.523252233	up
69576	Smco1	0.000664392	1.803720577	up
69581	Rhou	0.033335686	-2.039647577	down
69638	Enho	0.006054609	-1.301448171	down
69660	Tmbim1	0.027992355	-1.331336186	down
69684	Aarsd1	0.002970603	1.354863318	up
69710	Arap1	0.006455762	-1.206575342	down
69723	Rpain	0.018163187	1.269603098	up
69747	Zswim7	0.034149266	1.513032422	up
69806	Slc39a11	0.036594683	1.251980983	up
69821	Mterf4	0.006309726	1.390211046	up
69900	Mfsd11	0.001468102	-1.243350733	down
69917	Nabp2	0.016719306	-1.206323498	down
69923	Agk	0.042937483	1.225916454	up
69956	Ptcd3	0.02340737	1.445178881	up
69993	Chn2	0.049094659	-1.953488372	down
70031	Cmtm8	0.009427244	2.053302961	up
70240	Ufsp1	0.046347174	1.200856735	up
70297	Gcc2	0.015518455	1.634703196	up
70316	Ndufab1	0.008285772	1.40480163	up
70392	Asb12	0.039829095	1.403701851	up
70397	Tmem70	0.042818605	1.333911601	up
70456	Mpc2	0.008005813	1.491637767	up
70478	Mipep	0.034286861	1.236209335	up
70546	Zdhhc2	0.00588219	-1.809210526	down
70556	Slc25a33	0.046614409	1.584019204	up

70584	Pak4	0.000770912	-1.391136802	down
70681	Fam175a	0.01293403	1.305234534	up
70717	Medag	0.00580399	-2.48115942	down
70729	Nos1ap	0.049334202	-1.838187702	down
70772	Ggnbp1	0.023461485	1.374243258	up
70788	Klhl30	0.000100473	1.316268844	up
70804	Pgrmc2	0.007539127	-1.411109012	down
70873	Cnbd2	0.004746612	1.76872846	up
71093	Atoh8	0.009617837	-1.270845313	down
71147	Oxsm	0.00345184	1.336799538	up
71228	Dlg5	0.00209405	-1.471228616	down
71254	Naif1	0.012822116	-1.598253275	down
71323	Rassf8	0.026928739	-1.324106517	down
71365	Pdss2	0.007953597	1.333333333	up
71514	Sfpq	0.004313725	-1.268675123	down
71519	Cyp2u1	0.004845806	-1.431578947	down
71679	Atp5h	0.002765468	1.216112682	up
71687	Tmem25	0.021542585	1.841680129	up
71713	Cdc40	0.007450651	1.378910776	up
71770	Ap2b1	0.009727126	-1.203049203	down
71801	Plekhf2	0.006317647	1.319354839	up
71844	Nupl1	0.020556545	-1.233927607	down
71862	Gpr160	0.031504809	1.851851852	up
71886	2310002L09Rik	0.032891746	1.628303199	up
71890	Mad2l2	0.016240749	-1.259324419	down
71954	Suds3	0.026058071	-1.201007023	down
71978	Ppp2r2a	0.020715394	1.285126965	up
71984	Sars2	0.015357747	1.312187812	up
71989	Rpusd4	0.009220827	1.362995595	up
71994	Cnn3	0.014697306	-1.267963741	down
71998	Slc25a35	0.040331729	-1.334724541	down
72033	Tsc22d2	0.012011668	-1.441416894	down
72039	Mccc1	0.005355334	1.262476713	up
72054	Cyp4f18	0.025475019	2.238410596	up
72175	Mfsd8	0.038729311	1.312150838	up
72194	Fbxl20	0.006602095	1.295469049	up
72301	1810041L15Rik	0.046779776	-2.194029851	down
72345	Amer1	0.01821544	1.443181818	up
72350	Zc2hc1c	0.044307156	1.392307692	up
72397	Rbm12b1	0.02361085	-1.347663551	down
72416	Lrpprc	0.046875683	1.563857515	up
72421	Ttc30b	0.00888001	1.702325581	up
72479	Hsdl2	0.006910978	1.423770657	up
72480	Tspyl4	0.0466184	1.208160443	up
72549	Reep4	0.02381751	-1.372124493	down
72590	Ppme1	0.000937076	-1.27346217	down
72611	Zfp655	0.036112875	1.215865751	up
72658	2700097O09Rik	0.029288048	1.733791749	up
72668	Skida1	0.040198387	1.516528926	up

72739	Zkscan3	0.000664699	1.294440631	up
72759	Tmem135	0.00617502	1.458632532	up
72795	Ttc19	0.039962843	1.36394674	up
72805	Zfp839	0.030998562	1.242673993	up
72900	Ndufv2	0.004279733	1.394845964	up
73010	Gpr22	0.008186239	6.580952381	up
73095	Slc25a42	0.035080535	1.697979624	up
73139	Cenpv	0.02266375	1.424085576	up
73338	Itpripl1	0.02959693	1.324229075	up
73389	Hbp1	0.039746436	1.31517558	up
73451	Zfp763	0.00919302	2.333333333	up
73710	Tubb2b	0.027038888	-1.982222222	down
73724	Mcee	0.001139031	1.37847769	up
73728	Psd	0.044954705	-2.252307692	down
74011	Slc25a27	0.011670783	1.388372093	up
74019	Traf3ip1	0.028654172	-1.221271394	down
74100	Arpp21	0.026705191	2.917808219	up
74116	Pi16	0.010529124	-2.551906698	down
74143	Opa1	0.035949249	1.239798258	up
74147	Ehhadh	0.01804626	1.571088165	up
74158	Josd1	0.020993962	-1.266957921	down
74182	Gpcpd1	0.047221991	1.671590065	up
74204	Xpo6	0.032996083	1.258125472	up
74211	1700017B05Rik	0.002991269	-1.368758003	down
74238	Mterf2	0.03517385	1.521088435	up
74241	Chpf	0.003608751	-1.463571159	down
74246	Gale	0.04515907	-1.551181102	down
74318	Hopx	0.007387712	1.533298246	up
74320	Wdr33	0.037226117	1.257735779	up
74325	Cltb	0.003570446	-1.480200027	down
74365	Lonrf3	0.042365477	-2.826923077	down
74442	Sgms2	0.044362613	-1.953846154	down
74488	Lrrc15	0.026125547	2.679577465	up
74492	Kbtbd13	0.025750959	2.192090395	up
74617	Scpep1	0.022524561	-1.525849335	down
74747	Ddit4	0.019813615	-1.816037736	down
74761	Mxra8	0.006182154	-1.803559765	down
74775	Lmbr1l	0.048455103	-1.261127596	down
74776	Ppa2	0.032744651	1.376450528	up
74868	Tmem65	0.04385315	1.430625449	up
75029	Purg	0.005564089	-1.372881356	down
75216	Cep128	0.000778155	2.283333333	up
75219	Dusp18	0.023865572	1.531459988	up
75387	Sirt4	0.031877899	1.26994984	up
75420	Secisbp2	0.020013015	1.36159461	up
75530	Lyrm7	0.048653735	1.270637015	up
75564	Rsph9	0.007424218	-2.083798883	down
75573	Prr29	0.02365339	2.156521739	up
75581	Yipf7	0.0081864	1.640654206	up

75593	Malsu1	0.013390035	1.350103093	up
75692	Nr2c2ap	0.018803143	-1.338028169	down
75717	Cul5	0.026296265	1.437956204	up
75785	Klhl24	0.006922758	1.40028169	up
76089	Rapgef2	0.028163758	1.360503645	up
76187	Adhfe1	0.014353048	1.663123222	up
76246	Rtf1	0.004850043	1.231224397	up
76261	0610040J01Rik	0.023662349	2.615212528	up
76281	Tax1bp3	0.045185652	-1.256200625	down
76293	Mfap4	0.009278485	-4.05910683	down
76294	Asb5	0.048517382	1.837784372	up
76366	Mtif3	0.045847012	1.248271889	up
76467	Msrb2	0.010989525	1.239585492	up
76477	Pcolce2	0.001309634	-1.806595864	down
76577	Faf2	0.017387797	-1.259962049	down
76650	Srxn1	0.037193826	-1.321381837	down
76686	Clip3	0.018458495	-1.48694517	down
76722	Ckmt2	0.032424291	1.441124309	up
76826	Nubpl	0.037558552	1.384858044	up
76863	Dcun1d5	0.033127406	1.263077594	up
76933	Ifi27l2a	0.036643683	-1.738677094	down
76947	Ndufaf6	0.032785685	1.27343911	up
77006	Ddrgk1	0.004476872	-1.217711706	down
77057	Ston1	0.036453424	-1.442151805	down
77106	Tmem181a	0.000242669	1.632247143	up
77128	Crebrf	0.044041374	1.410649819	up
77552	Shisa4	0.004435897	-2.058031088	down
77559	Agl	0.003079183	1.231258967	up
77578	Bcl9	0.04312435	1.386378962	up
77579	Myh10	0.008918518	-1.366799205	down
77622	Apex2	0.005510835	2.083098592	up
77652	Zfp955a	0.04878316	1.498322148	up
77864	Ypel2	0.007982643	-1.606604451	down
78038	Mccc2	0.002497059	1.553359684	up
78070	Cpt1c	0.038961556	-1.47826087	down
78330	Ndufv3	0.014198589	1.34849235	up
78334	Cdk19	0.006994411	1.345333333	up
78394	Ddx52	0.04760126	1.374774775	up
78541	Asb8	0.02308799	1.267898782	up
78688	Nol3	0.036949086	-1.272102921	down
78749	Filip1l	0.007308292	-1.350819672	down
78754	Galnt15	0.000359249	1.710776942	up
78785	Clip4	0.002094152	-1.345034642	down
78802	Ttc30a1	0.002983153	1.619377163	up
78834	Zfp623	0.009625461	-1.382940109	down
78890	Trmt44	0.045207032	-1.470119522	down
78892	Crispld2	0.00054257	-2.054478301	down
78926	Gas2l1	0.040794661	-1.54856941	down
78935	Saal1	0.03582354	-1.379732739	down

79202	Tnfrsf22	0.023625942	1.468062827	up
80748	BC004004	0.005316385	1.216476944	up
80752	Fam20c	5.22222E-05	-1.353652128	down
80860	Ghdc	0.015582623	-1.433651344	down
80876	Ifitm2	0.021220464	-1.473604177	down
80877	Lrba	0.011563582	1.254749148	up
80879	Slc16a3	0.001741418	-1.69451074	down
80891	Fcrls	0.008191433	-1.705801105	down
80903	Fgf16	0.00482714	3.570038911	up
80907	Lactb	0.018299595	1.367015099	up
81013	Vmn1r65	0.029080437	3.2	up
81014	Vmn1r58	0.026568817	1.360033031	up
81703	Jdp2	0.031159625	-1.728976697	down
81904	Cacng7	0.012483145	-1.394195889	down
83396	Glis2	0.012587702	-1.949502678	down
83431	Ndel1	4.55731E-05	-1.407666521	down
83433	Trem2	0.02829006	-2.053884712	down
83456	Mov10l1	0.007479842	1.612073293	up
83554	Fstl3	0.001117302	-2.802298851	down
83797	Smarcd1	0.005355573	1.317927171	up
93679	Trim8	0.025082357	-1.23237998	down
93723	Pcdhga11	0.039267996	-1.921708185	down
93734	Mpv17l	0.029072166	1.552356021	up
93747	Echs1	0.03343466	1.247324946	up
93888	Pcdhb17	0.030341053	-1.304849885	down
93893	Pcdhb22	0.032075961	-1.776119403	down
94065	Mrpl34	0.010868843	1.292201853	up
94089	Trim7	0.007530233	2.73198594	up
94185	Tnfrsf21	0.048467378	1.424266455	up
94187	Zfp423	0.036335287	-1.468879668	down
97130	C77080	0.049739331	-1.595854922	down
97387	Strn4	0.010024647	-1.23812132	down
98402	Sh3bp4	0.017054938	-1.393442623	down
99010	Lpcat4	0.034676271	-1.579349904	down
99138	Stard7	0.02143415	1.30965502	up
99412	Golga2	0.041143569	-1.247159091	down
99543	Olfml3	0.001165918	-1.839375	down
100017	Ldlrap1	0.014638527	-1.284131206	down
100129	Gpr153	0.001515565	-2.174944812	down
100952	Emilin1	0.010423304	-1.561628555	down
101197	Zfp956	0.032345238	1.356466877	up
101631	Pwwp2b	0.04933863	-1.443734015	down
101883	Igflr1	0.025132529	1.324369748	up
102032	Smim19	0.008822209	1.207474519	up
102632	Acad11	0.012092985	1.259630093	up
103583	Fbxw11	0.025240502	-1.221811862	down
104111	Adcy3	0.016993599	-1.375308642	down
104394	E2f4	0.006576447	-1.201584669	down
104776	Aldh6a1	0.0073847	1.391931034	up

104923	Adi1	0.03889008	1.288248337	up
106344	Rfc4	0.047013606	1.417601381	up
106522	Pkdcc	0.02438182	-1.606666667	down
106564	Ppcs	0.017756564	1.226571767	up
106672	AI413582	0.024642112	-1.695884774	down
106759	Ticam1	0.017760413	-1.345289079	down
106869	Tnfaip8	0.009706399	2.685765443	up
107733	Mrpl41	0.044259293	1.205174687	up
107817	Jmjd6	0.036614304	-1.235342153	down
107869	Cth	0.033244753	2.986013986	up
108014	Srsf9	0.005359174	-1.2171733	down
108075	Ltbp4	0.002702993	-1.659646719	down
108100	Baiap2	0.015856852	-1.565065502	down
108116	Slco3a1	0.030730966	-1.561674628	down
108153	Adamts7	0.009598493	1.763245033	up
108155	Ogt	0.029192142	1.233867735	up
108654	Fam210a	0.003189457	1.398098771	up
108707	Fam207a	0.025162242	-1.269789227	down
108853	Mtrf1l	0.000299163	1.641447368	up
109042	Prkcdbp	0.019120019	-1.425440613	down
109136	Mmaa	0.03854239	1.287096013	up
109154	Mlec	0.032853462	-1.29839374	down
109222	Rarres1	0.032751804	1.679775281	up
109245	Lrrc39	0.024893351	1.496974063	up
109754	Cyb5r3	0.001919183	-1.398320011	down
109900	Asl	0.013316967	-1.308605341	down
110213	Tmbim6	0.025207647	1.281669337	up
110253	Triobp	0.005828067	-1.251542477	down
110385	Pde4c	0.028200092	1.834042553	up
110391	Qdpr	0.02771968	1.221013926	up
110446	Acat1	0.017580952	1.425112844	up
110821	Pcca	0.002894786	1.48520638	up
110826	Etfb	0.011509399	1.578137951	up
110829	Lims1	0.043255275	1.323100246	up
110842	Etfa	0.009522501	1.659766703	up
112405	Egln1	0.049389015	1.203952588	up
114663	Impa2	0.019685504	1.864344638	up
117589	Asb7	0.048434299	1.2448	up
117599	Helb	0.015414182	1.316596932	up
118445	Klf16	0.021515219	-1.362723214	down
140494	Atp6v0a4	0.028677315	-6.155555556	down
140795	P2ry14	0.007172945	1.871143376	up
142687	Asb14	0.000458089	1.410253887	up
170643	Kirrel	0.001764136	-1.487283825	down
170718	Idh3b	0.039764757	1.357193603	up
170728	Rtn4ip1	0.040573237	1.285012566	up
170829	Tram2	0.004446758	-1.66487214	down
170835	Inpp5j	0.009998697	-1.403210273	down
171095	Il17rc	0.011777223	-1.342504744	down

171171	Ntng2	0.007366601	-1.853641457	down
171210	Acot2	0.042495866	2.039424404	up
171508	Creld1	0.001642318	-1.216664285	down
171543	Bmf	0.003459091	1.610778443	up
171580	Mical1	0.028958742	-1.229012346	down
192156	Mvd	0.040904378	1.289473684	up
192166	Sardh	0.003215425	-1.536585366	down
192173	Fam195b	0.012255592	-1.322460292	down
192289	Tmlhe	0.012490124	1.348655681	up
192976	Lrrc75a	0.032551646	-1.908333333	down
207165	Bptf	0.038936203	-1.292347377	down
207565	Camkk2	0.015686729	-1.261927035	down
208228	Mob3a	0.032245646	-1.670711974	down
208650	Cblb	0.013071685	1.289795918	up
208922	Cpeb3	0.012805108	1.602996255	up
208943	Myo5c	0.003263567	2.664634146	up
208968	Zfp280c	0.036940837	1.490774908	up
209047	Gipc3	0.024500055	1.884557721	up
209212	Osgin2	0.02231529	1.380462725	up
209378	Itih5	0.005281564	-1.884026258	down
209692	Dhtkd1	0.009760592	1.542857143	up
209760	Tmc7	0.011859362	1.601202405	up
209773	Dennd2a	0.014341033	-1.608617594	down
210004	B3gntl1	0.003058279	1.450317125	up
210106	Papd7	0.03180492	-1.225517627	down
210622	Pamr1	0.001013549	-2.974235105	down
210710	Gab3	0.029508712	2.09	up
211064	Alkbh1	0.031525678	-1.382124352	down
211134	Lzts1	0.02507938	1.51980198	up
211253	Mtrf1	0.019469716	1.430934657	up
211548	Nomo1	0.013162358	-1.279834445	down
212398	Frat2	0.005666116	2.254807692	up
212933	Pm20d1	0.015087974	1.549872123	up
212943	Fam46a	0.005265029	-1.741248097	down
213391	Rassf4	0.025397409	1.355337079	up
213945	Col28a1	0.040682031	-2.048780488	down
214133	Tet2	0.00612263	1.481069042	up
214239	A430105I19Rik	0.003129633	1.416926921	up
214254	Nudt15	0.045843696	1.286329386	up
214305	Hhipl1	0.02439162	-1.777292576	down
214498	Cdc73	0.016126145	-1.286814244	down
214579	Aldh5a1	0.003761217	1.597122302	up
214580	Pstk	0.008567981	1.320495186	up
214742	Rcor3	0.044117575	1.205599666	up
214812	Zfp609	0.045645259	-1.25516834	down
216001	Micu1	0.049249469	-1.262402496	down
216148	Shc2	0.012637305	-1.345609065	down
216198	Tcp11l2	0.002137622	1.506760411	up
216705	Clint1	0.01313439	-1.247294195	down

216725	Adamts2	0.020276197	-2.000628931	down
216767	Mrpl22	0.008297293	1.405585106	up
216831	Arhgap44	0.020001078	-1.352919971	down
216856	Nlgn2	0.002588226	-1.733766234	down
216858	Kctd11	0.003694033	-1.926509186	down
216860	Neurl4	0.049044183	1.20548806	up
216869	Arrb2	0.017788762	-1.213570982	down
217124	Ppp1r9b	0.015844072	-1.426605505	down
217125	Samd14	0.038841515	-1.532646048	down
217198	Plekhh3	0.01825363	1.338582677	up
217219	Fam171a2	0.046209749	-1.438976378	down
217258	Abca8a	0.015712922	-1.339770554	down
217262	Abca9	0.003896863	-1.416068867	down
217333	Trim47	0.02896288	-1.697765492	down
217370	BC017643	0.006574239	-1.361097257	down
217430	Pqlc3	0.006196113	-2.023929471	down
217473	Ankmy2	0.021460927	1.239430352	up
217615	Ctage5	0.009867851	1.276540284	up
217666	L2hgdh	0.037020337	1.277425204	up
217707	Coq6	0.042278964	1.24557976	up
217734	Pomt2	0.019676689	-1.285035629	down
217827	Nrde2	0.011665597	1.386690647	up
217864	Rcor1	0.037734989	-1.200448766	down
217882	Cep170b	0.010846231	-1.379069767	down
217980	Larp4b	0.028125825	1.200996678	up
218454	Lhfpl2	0.008072681	-2.102362205	down
218461	Pde8b	0.024351518	1.710884354	up
219072	Haus4	0.010450019	1.383193277	up
223272	Itgbl1	0.021562124	-3.086251067	down
223642	Zc3h3	0.040115992	-1.223942208	down
223693	Tmem184b	0.037987519	-1.251945989	down
223722	Mcat	0.029994544	1.330306088	up
223881	Rnd1	0.027457829	-2.370535714	down
224024	Scarf2	0.003170872	-1.624356775	down
224405	Cyyr1	0.018271516	1.927781013	up
224440	Setd4	0.035053442	1.720930233	up
224697	Adamts10	0.011183229	-1.578216374	down
225010	Lclat1	0.039447132	1.819113127	up
225049	Ttc7	0.02057385	1.3010279	up
225215	Rsl24d1	0.020744035	1.294916807	up
225326	Pik3c3	0.015462158	1.230832988	up
225339	Ammecr1l	0.033850199	-1.221585482	down
225861	Snx32	0.03158817	-1.37398374	down
225887	Ndufs8	0.016211167	1.25541472	up
226144	Erlin1	0.009726759	-1.333901193	down
226169	Pprc1	0.019997096	-1.35023989	down
226519	Lamc1	0.027313597	-1.295422606	down
226525	Rasal2	0.00640642	1.318489835	up
226791	Lyplal1	0.039507236	1.662698413	up

226977	Actr1b	0.040718134	-1.263023865	down
227197	Ndufs1	0.027845484	1.330182212	up
227399	Ppip5k2	0.011989675	1.937098845	up
227638	Qsox2	0.003654964	1.678977273	up
227674	Ddx31	0.0380648	-1.301339286	down
227731	Slc25a25	0.037436902	-1.387596899	down
227737	Fam129b	0.039251962	-1.486194478	down
228033	Atp5g3	0.015864178	1.339331646	up
228136	Zdhhc5	0.007945326	-1.265110032	down
228564	Frmd5	0.001999666	-1.810924739	down
228576	Mall	0.006138089	-1.691602317	down
228662	Btbd3	0.010947396	1.297101449	up
228785	Mylk2	0.044874334	-3.432432432	down
228836	Dlgap4	0.016824966	-1.245146014	down
228966	Ppp1r3d	0.001934392	1.5	up
229487	Gatb	0.042516228	1.358727245	up
229599	Ciart	0.025469666	3.087824351	up
229905	Ccbl2	1.49294E-06	1.529062577	up
230027	Coq3	0.025612311	1.650665503	up
230088	Fam214b	0.004315713	-1.226526892	down
230649	Atpaf1	0.037005831	1.270432178	up
230657	Tmem69	0.001351358	1.332544633	up
230837	Asap3	0.046983866	1.511022044	up
230967	Cep104	0.043966124	-1.244147157	down
231003	Klhl17	0.006989316	1.264650284	up
231042	Nupl2	0.009789702	1.428783383	up
231086	Hadhb	0.031965141	1.559692868	up
231151	Tada2b	0.027855916	-1.210759845	down
231329	Polr2b	0.03214655	1.214482586	up
231637	Ssh1	0.032448384	-1.352735739	down
231858	Radil	0.025462717	-1.488095238	down
232431	Gprc5a	0.004510489	-2.554054054	down
232536	Mrps35	0.039351086	1.327221597	up
232664	Ccdc136	0.003622593	-2.731404959	down
232679	Zc3hc1	0.000392126	1.217800289	up
232816	Zfp628	0.024626739	-1.203125	down
232906	Arhgap35	0.03656562	-1.23937247	down
232969	Zfp428	0.031349484	1.559549589	up
233011	Itpkc	0.032478992	-1.278959811	down
233033	Samd4b	0.049985138	-1.436967632	down
233107	Kctd15	0.005338672	-2.15234375	down
233168	AI987944	0.043242194	1.421652422	up
233328	Lrrk1	0.018925659	-1.484210526	down
233489	Picalm	0.032249856	-1.377000539	down
233571	P2ry6	0.017844395	-1.358856089	down
233902	Fbxl19	0.012349205	-1.550255537	down
233908	Fus	0.004211352	1.378475664	up
234577	Cpne2	0.024744948	-1.321933962	down
234725	Zfp612	0.005887983	1.741176471	up

234839	Piezo1	0.012310866	-1.308210391	down
235283	Gramd1b	0.049379033	1.586419753	up
235493	Fam214a	0.004115162	1.68125855	up
235611	Plxnb1	0.009220011	1.706763036	up
236920	Stard8	0.011211754	1.242230347	up
237353	Sh3rf3	0.023026834	-2.402439024	down
237403	Lingo3	0.000176339	3.563380282	up
237754	Btnl9	0.039414476	1.883216783	up
237775	Zfp867	0.025809667	1.523722628	up
237988	Cdr2l	0.017944253	-1.321688501	down
238123	Cog5	0.007856829	1.323937153	up
238276	Akap5	0.032243196	2.294117647	up
239017	Ogdhl	0.036162327	2.272501886	up
239719	Mkl2	0.009186194	1.266828381	up
240064	Zfp799	0.013005912	1.441717791	up
240084	Cchcr1	0.04744575	-1.226487524	down
240660	Slc35g1	0.041887526	1.535384615	up
240752	Pik3c2b	0.043562609	1.289026275	up
243219	2900026A02Rik	0.039777183	-1.281726619	down
243371	Lrrc61	0.033669641	1.22592068	up
243382	Ppm1k	0.0078684	1.971399387	up
243653	Clec1a	0.01765367	1.797188755	up
243905	Zfp568	0.026573132	-1.69130788	down
244152	Tsku	0.045755399	-1.733624454	down
244329	Mcph1	0.034598862	1.344	up
244556	Zfp791	0.026793765	1.553072626	up
244864	Layn	0.015106418	-1.589519651	down
245038	Dclk3	0.035053113	-5.709677419	down
245350	AA414768	0.046869577	-1.621890547	down
245404	Dcaf12l1	0.018805517	2.358139535	up
246196	Zfp277	0.039578163	1.237320945	up
246228	Vwa1	0.017609882	-1.626908203	down
246738	Dnajc28	0.006531632	1.689530686	up
260297	Prrt1	0.02405776	-1.661577608	down
263406	Plekhg3	0.0203322	-1.346497765	down
268297	Scml4	0.012919793	-2.52173913	down
268396	Sh3pxd2b	0.022409065	-1.945578231	down
268445	Ankrd13b	0.000421835	-1.437400951	down
268739	Arhgef40	0.002271351	-1.723770492	down
269060	Dagla	0.006654856	-2.248648649	down
269397	Ss18l1	0.014633925	1.395778364	up
269423	Abhd18	0.019901265	1.557660626	up
269593	Luzp1	0.012560275	-1.239915966	down
269713	Clip2	0.003635199	-1.869029276	down
269717	Orai2	0.019427791	-1.920930233	down
269941	Chsy1	0.000324388	-1.268518519	down
270076	Gcdh	0.000931869	1.470794521	up
270166	Clpx	0.034158975	1.708713432	up
270192	Rab6b	0.031593283	-1.576441103	down

271981	Tbck	0.028361109	1.205761317	up
272322	Arntl2	0.008497396	2.295180723	up
272396	Tarsl2	0.031120824	1.282557454	up
277396	Klhl23	0.009633685	1.524066577	up
277743	Fam131c	0.041054786	-1.985074627	down
278097	Armcx6	0.011511227	-2.066350711	down
280408	Rilp	0.040429629	1.386937847	up
280668	Adam1a	0.035699079	1.547112462	up
319262	Fchsd1	0.049324225	-1.321875	down
319604	Fam168a	0.028869348	-1.213806328	down
319613	Sybu	0.001073597	2.067001675	up
319653	Slc25a40	0.024633871	1.465558195	up
319675	Cep295	0.047154411	1.423127464	up
319845	Bbs9	0.009237899	-1.310866575	down
319953	Ttll1	0.01551726	2.084858128	up
319998	Tmem198	0.044874334	-2.097560976	down
320024	Nceh1	0.010804679	1.362632046	up
320078	Olfml2b	0.043789214	-2.492931197	down
320487	Heatr5a	0.021433004	-1.271804062	down
320502	Lmod3	0.022827283	1.556241581	up
320720	Fastkd1	0.011002942	1.359351988	up
320878	Mical2	0.030387487	-1.494425928	down
321000	Lrif1	0.005607665	1.478097622	up
328035	Fads6	0.037613558	-1.929824561	down
328232	Gfod1	0.04647618	-1.433201927	down
328949	Mcc	0.011763374	1.366564417	up
329154	Ankrd44	0.012028715	1.514851485	up
329384	Ptrh1	0.029537422	-1.43697479	down
329470	Accs	0.025578863	1.425339367	up
330064	Slc5a6	0.043378946	1.3570939	up
330171	Kctd10	0.02448651	-1.250092308	down
330177	Taok3	0.000562953	-1.658503401	down
330409	Cecr2	0.013955522	2.032258065	up
331524	Xkrx	0.005104864	1.885321101	up
331623	Bend3	0.021620426	1.525616698	up
338359	Supv3l1	0.000873706	1.234398496	up
338364	Trim65	0.025576924	-1.409302326	down
353169	Slc2a12	0.032697889	1.495283019	up
378702	Serf2	0.031540949	-1.275979136	down
380608	Tagap1	0.008581748	1.46286031	up
380755	Lsmem1	0.024677787	1.458233317	up
380840	Lyrm4	0.045056737	1.252009	up
380928	Lmo7	0.004850674	1.308508358	up
380969	Nckap5l	0.016782664	-1.426307448	down
381259	Tmem237	0.037945527	-1.374789916	down
381493	S100a7a	0.029921805	-3.757575758	down
381582	Tmem240	0.025753181	-11.38461538	down
381801	Tatdn2	0.012117749	-1.215145699	down
382051	Pdp2	0.003727514	3.545787546	up

382056	Crtc1	0.036800783	-1.395765472	down
382137	Fdxacb1	0.028800947	1.543887147	up
382562	Pfn4	0.003074119	1.496845426	up
384009	Glipr2	0.030909602	-1.419191919	down
407785	Ndufs6	0.02920988	1.481863084	up
407800	Ecm2	0.016965892	-1.218093093	down
432442	Akap7	0.044990883	1.330927052	up
432779	Lrrc14b	0.000210974	1.489182692	up
433375	Creg1	0.01018121	1.359361593	up
433771	Minos1	0.041685532	1.288964466	up
434179	Gm5595	0.002889654	2.724351051	up
434215	Lrrc32	0.002041131	-1.672738312	down
434233	Gm5601	0.013169267	-1.474293497	down
434437	Amt	0.011102394	1.223013049	up
435766	Tnni3k	0.031681562	1.258080077	up
436022	Dnaaf3	0.004646372	2.120521173	up
504186	Chrna10	0.04148007	1.497175141	up
545276	Gal3st3	0.021425326	2.201654602	up
546611	Klhl33	0.045048362	2.20970266	up
595136	Ndufs5	0.024913206	1.26889403	up
619597	Gm6086	0.022988849	2.775510204	up
622675	Zfp827	0.031353777	1.350857143	up
623661	Lipt1	0.020396814	1.453081232	up
625360	BC147527	0.023487245	3.333333333	up
628308	Gm14420	0.044904688	1.402714067	up
629059	Fam124a	0.003382346	-1.484808454	down
629378	Dact3	0.003996641	-1.598143852	down
630751	LOC630751	0.008367188	42.05882353	up
633640	Gm7120	0.004338625	2.069613644	up
641340	Nrbf2	0.003160394	1.397323726	up
654801	Zfp784	0.018402591	1.239929947	up
666642	Gm8210	0.023383042	2.692874693	up
666907	Ms4a4a	0.032254858	-1.645283019	down
673094	Cd99	0.017638639	-1.442165914	down
100041085	Mfsd4b3	0.015260856	1.529767911	up
100041194	Ahnak2	0.018851231	-2.347266881	down
100041677	Gm13157	0.040118137	1.539473684	up
100169864	Gm44504	0.022735031	-3.90625	down
100862085	Gm16867	0.024942	-3.782608696	down
100862375	Gm21685	0.018866117	-2.10135705	down
101056102	Gm29779	0.015967549	-1.751992914	down
101056408	Gm15816	0.039794625	-1.565181518	down
102635879	LOC102635879	0.035460271	-3.975	down
102638515	LOC102638515	0.007374279	-2.060402685	down
105242736	LOC105242736	0.008429726	4.384615385	up
105242869	Gm38958	0.035787655	-2.355263158	down
105245547	Gm40991	0.02718331	2.465838509	up
105246811	Gm42035	0.014170732	-1.270011274	down
105247050	Gm42226	0.001624631	-2.914563107	down

108167440	0.026550431	2.835497835	up
108167858	0.002867162	-1.675866496	down
108167951	0.047102088	-2.559055118	down
108168101	0.036412801	1.560627675	up
108168376	0.004654861	-1.694834617	down
108168747	0.038418542	1.38079096	up
108169043	0.004009376	1.349820949	up
108169061	0.032539356	2.00174216	up
108169093	0.000631354	2.688663283	up
